# Extreme Heat and Emergency Health Impacts in the US (2018–2025)

**DOI:** 10.3390/ijerph23081074

**Published:** 2026-08-18

**Authors:** Tyler Hecht, Baoyuan Zhou, Abhi Thanvi, Lelys Bravo de Guenni

**Affiliations:** Department of Statistics, University of Illinois Urbana-Champaign; Champaign, IL 61820, USA; thecht2@illinois.edu (T.H.); baoyuan2@illinois.edu (B.Z.); abhithanvi007@gmail.com (A.T.)

**Keywords:** emergency department visits, heat index, extreme temperature, mixed effects models

## Abstract

**Highlights:**

**Public health relevance—How does this work relate to a public health issue?**

- Extreme heat is a major and increasing public health hazard linked to rising emergency department visits in the United States.

**Public health significance—Why is this work of significance to public health?**

- The study quantifies real-time and regionally varying health impacts of extreme heat, showing that both temperature and heat index significantly predict emergency department visits.

**Public health implications—What are the key implications or messages for practitioners, policy makers and/or researchers in public health?**

- Findings support early heat-health interventions and region-specific response strategies, emphasizing the need for real-time monitoring and improved preparedness to reduce heat-related health burdens.

**Abstract:**

Future climate projections suggest an increase in heat-related mortality and a decrease in cold-related deaths under warming scenarios. Understanding the health impacts of extreme heat, and their implications for healthcare demand is essential for assessing the future burden of climate-related illnesses. In this study, we examined the relationship between extreme heat events and Emergency Department Visits (EDV) for heat-related illnesses (HRIs) across the United States from 2018 to 2025. Using data from the Centers for Disease Control and Prevention (CDC) Heat and Health Tracker and other relevant sources, we analyzed EDV rates standardized to 100,000 population. We aggregated daily into the 10 U.S. Health and Human Services (HHS) Regions. We used 0.5° × 0.5° gridded maximum daily temperature data (aggregated to HHS regions with proportional area weighting) and daily maximum heat index extracted from the CDC data portal (estimated using the US National Weather Service methodology and aggregated to HHS regions using total population weighting) to characterize seasonal patterns and regional variability. The association between peak heat events and EDV time series was explored using log-linear mixed-effects models, which accounted for seasonal trends, climate variables, and their regional variability. Random effects were used to capture regional heterogeneity in predictor-response relationships, accommodating variation in associations across regions. Model performance was evaluated using prediction error metrics and goodness-of-fit assessments. Maximum temperature and heat index were both significant predictors, with the heat index offering a slightly better fit. Associations were largely contemporaneous, with peak correlations at lag zero, underscoring the need for real-time response. EDV increased several days before peak environmental conditions, consistent with early exposure effects. While temperature-EDV relationships varied regionally, heat index associations were more stable. This work underscores the urgent need for regionally adaptive public health strategies in the face of intensifying climate extremes and outlines future directions for research and policy to strengthen health systems’ preparedness in a warming world.

## 1. Introduction

Extreme heat events stand out as one of the most deadly weather-related hazards worldwide. Heat-related events caused the most death in the U.S. during 2023, followed by wildfires and tornadoes. The largest number of reported injuries during that year were caused by heat, tornadoes and winter weather [1].

During the period 2022–2025, 41 countries faced national breaking temperature records [2]. Five million deaths were attributed to extreme heat and cold globally between 2000–2019 [3], and projections indicate that heat-related deaths will increase.

Climate change might decrease cold-related deaths at high latitudes but increases heat-related deaths especially in Tropical Africa and Asia [4].

Impacts of these events can extend beyond individual increased mortality and morbidity to challenging the resilience and response of the health care system.

Ref. [5] identified a broad spectrum of heat-related diseases including electrolyte imbalances, cardiovascular disease and mental health issues. Using recent surveillance data, ref. [6] report substantial increases in heat-related emergency department visits across the United States, with pronounced regional and demographic variability, underscoring the growing public health burden of extreme heat.

Ref. [7] documented increasing heat-related mortality across nearly all U.S. climate regions between 1981 and 2022. The study identified the number of extreme heat days as the strongest predictor of heat-related mortality and suggested that the United States may be experiencing a new, elevated baseline of heat-related mortality. Similarly, ref. [8] found that heat waves were associated with substantial increases in annual mortality among older adults in the United States, estimating more than 17,000 excess deaths attributable to heat-wave exposure. Their findings also highlighted important disparities, with stronger effects observed among Black populations and residents of high-poverty neighborhoods, while greater neighborhood green space appeared to confer protection.

These recent findings are consistent with a large body of epidemiological research demonstrating associations between high temperatures, heat indices, and a range of adverse health outcomes, including mortality, hospitalizations, and emergency department visits. Previous studies have characterized exposure-response relationships at county, city, national, and international scales using advanced analytical approaches such as distributed lag nonlinear models, generalized additive models, and spatiotemporal models. Collectively, this literature has substantially advanced understanding of heat-health relationships and revealed marked geographic differences in heat-related vulnerability.

Understanding the impacts of extreme heat on health and health services demand would provide a better estimation of future climate-related illness burden. While numerous studies evaluate associations between heat metrics and health outcomes, fewer have explicitly investigated the temporal synchronization between peaks in environmental exposures and peaks in healthcare utilization. Understanding whether and where extreme heat events coincide with surges in emergency department demand is critical for improving preparedness, resource allocation, and the effectiveness of early-warning systems. The inclusion of recent years characterized by record-breaking temperatures provides an opportunity to reassess the heat-health relationship under climatic conditions that increasingly resemble those expected in the future.

In this paper we focus on evaluating the synchronicity between ambient variables peak timing and emergency department visits peak timing in the large contiguous US states by Health and Human Services (HHS) regions building predictive models for Emergency Department Visits (EDV) that account for regional impacts of environmental factors as well as geographical factors. In Section 2 we provide a description of all data sources used in the analysis. In Section 3 we describe the methodology including the different modeling approaches for seasonality quantification and predictive models for EDV across the 10 HHS regions. Results are provided in Section 4 including model diagnostics and predictive skills. Discussion and Conclusions are presented in Section 5 and Section 6 with further discussions on the limitations of this approach and future research directions.

## 2. Data Description

Data for this research was extracted from different sources. Data pre-processing was necessary for consistency and compatibility of different temporal and spatial scales. This section describes the different data sources and data processing for environmental variables, as well as the health related outcome of this research which is Emergency Department Visits.

### 2.1. Emergency Department Visits

**Figure 1:**
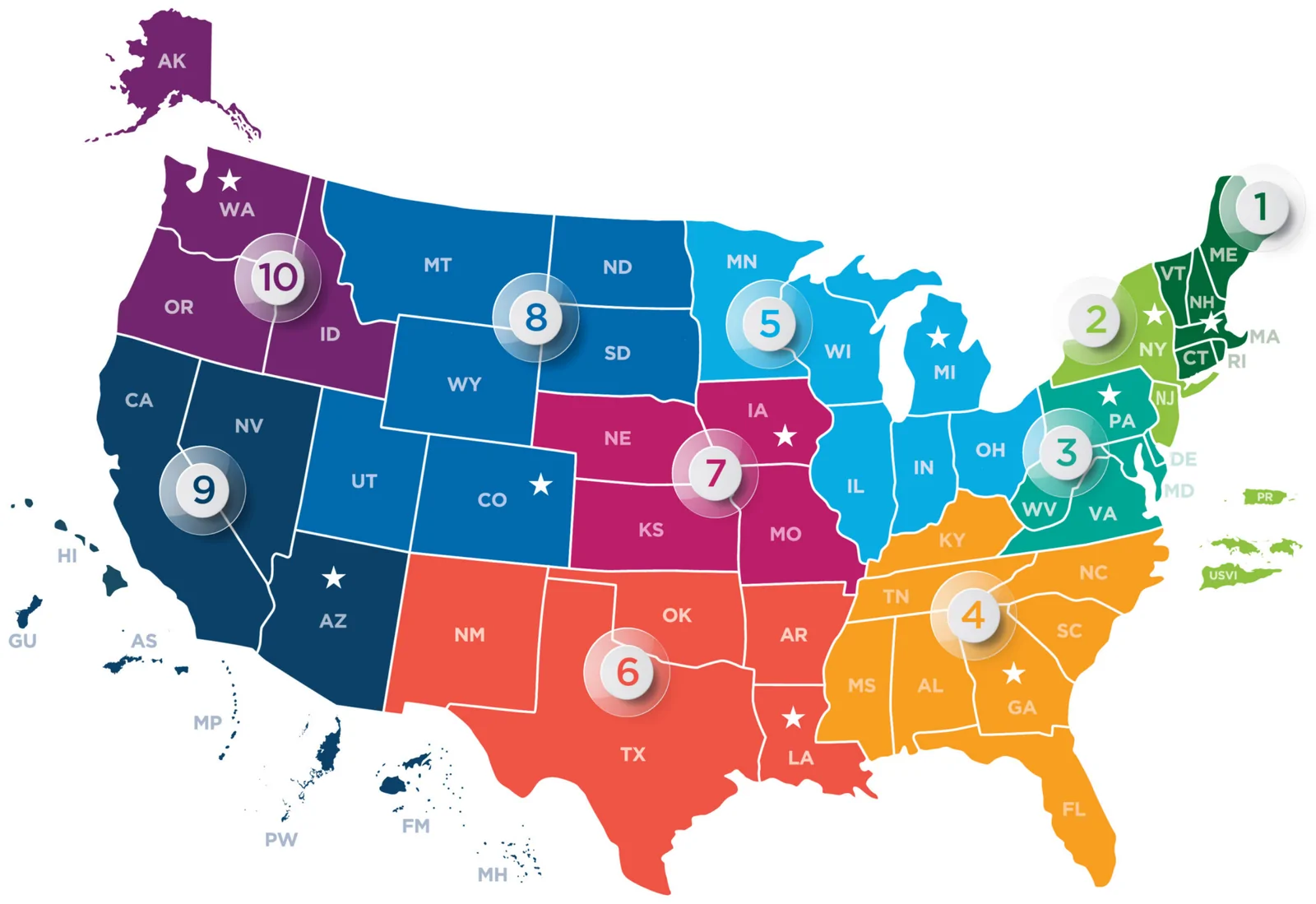
The ten Health and Human Services regions represented by the different colors and numbers on the map. Stars indicate the location of the main regional offices. Image taken from https://r4phtc.org/phtcn/#maps (accessed on 17 February 2025).

The emergency department visits (EDV) data was obtained from the Centers for Disease Control and Prevention’s (CDC’s) National Environmental Public Health Tracking Network (https://ephtracking.cdc.gov/DataExplorer/, accessed on 24 February 2026) under the heat-related illness (HRI) content. Daily data from 1 January 2018 to 31 December 2025 was used in this study for each of the ten Health and Human Services (HHS) regions (see Figure 1). EDV was expressed as HRI-associated visits per 100,000 emergency department visits.

Because EDV data was only available by HHS region, the temperature and heat index data were aggregated to this level. Furthermore, because the EDV data did not extend further back than 2018 (other than 31 December 2017, which was excluded for simplicity), temperature data was limited to this range despite being available at prior years.

### 2.2. Maximum Temperature Data

The temperature data was obtained from the Physical Sciences Laboratory of the National Oceanic and Atmospheric Administration (NOAA) (https://psl.noaa.gov/data/gridded/data.cpc.globaltemp.html, accessed on 3 February 2025). The maximum surface temperature in Celsius at a daily rate from 1 January 2018 to 31 December 2025 was used. Because the data was gridded in 0.5 × 0.5 degree latitude and longitude squares, it needed to be aggregated to the HHS regional level.

Shape-files from the United States Census Bureau (https://www.census.gov/geographies/mapping-files/time-series/geo/carto-boundary-file.html, accessed on 4 March 2025) were used at the 500 km resolution level. The daily temperature for each region was calculated by averaging the gridded values, weighted by the proportion of the area in the region to account for grid overlapping between two regions.

### 2.3. Heat Index

Heat Index (HI) data were obtained from the CDC’s National Environmental Public Health Tracking Network, specifically from the “HRI” section and the “Historical Temperature and Heat Index” subsection. These data are available at a daily resolution at the county level for the period 1 January 2018 through 31 December 2022, and were additionally aggregated to the HHS regional level.

The heat index integrates both, the air surface temperature and the relative humidity, and it is frequently used to measure heat exposure [9]. For calculating the Heat Index, CDC uses a National Weather Service’s modified version of the Rothfusz regression (National Weather Service, “The Heat Index Equation,” https://www.wpc.ncep.noaa.gov/html/heatindex_equation.shtml, accessed on 4 March 2025).

(1) $$\begin{array}{cc} HI= & -42.379+2.04901523\;T+10.14333127\;RH-0.22475541\;T\times RH\\ \; & -6.83783\times 10^{-3}T^{2}-5.481717\times 10^{-2}RH^{2}\\ \; & +1.22874\times 10^{-3}T^{2}\times RH+8.5282\times 10^{-4}T\times RH^{2}\\ \; & -1.99\times 10^{-6}T^{2}\times RH^{2} \end{array}$$

where HI is heat index (°F); *T* is the air temperature (°F) and RH is the relative humidity expressed as a percentage (%). The raw data are collected daily at the county level across all 3143 US counties and sourced from the CDC website.

A two-step aggregation procedure was used to construct HHS regions heat indices. County-level values were first averaged within states to prevent states with a larger number of counties from disproportionately influencing regional estimates. State-level heat indices were then aggregated using population weights so that the resulting regional measure reflects the distribution of residents across states within each HHS region. The detailed calculations follow:

- First, compute the average heat index for each state:
  (2) $$\begin{array}{cc} {\mathrm{HI}}_{\mathrm{state}}= & \frac{1}{n_{c}}\sum_{i=1}^{n_{c}}{\mathrm{HI}}_{{\mathrm{county}}_{i}} \end{array}$$
  where $n_{c}$ is the number of counties in the state.
- Then, calculate the population-weighted average heat index for each HHS region:
  (3) $$\begin{array}{cc} {\mathrm{HI}}_{\mathrm{region}}= & \frac{\sum_{j\in \mathrm{region}}{\mathrm{HI}}_{{\mathrm{state}}_{j}}\times {\mathrm{Pop}}_{j}}{\sum_{j\in \mathrm{region}}{\mathrm{Pop}}_{j}} \end{array}$$
  where ${\mathrm{Pop}}_{j}$ is the total population for state *j* and ${\mathrm{HI}}_{\mathrm{state}j}$ is the heat index for state *j* calculated from Equation (2), and ${\mathrm{HI}}_{\mathrm{region}}$ is the weighted average heat index by region.

### 2.4. Vulnerability-Related Variables

Vulnerability-related data were obtained from the CDC’s National Environmental Public Health Tracking Network under the “HRI” (heat-related illnesses) Section, “Vulnerability and Preparedness: Heat” subsection. These variables capture demographic and environmental characteristics that may influence population susceptibility to HRI. Three variables were considered in the analysis:

- elderly_percentage: Percentage of the population aged 65 or older living alone. This variable is collected annually at the state level from 2009 to 2022.
- nonwhite_percentage: Percentage of the population identifying as a race other than White. This variable is available at the state level for the year 2020.
- forest_coverage: Percentage of land area covered by forest. This variable is measured every five years at the state level from 2001 to 2021.

## 3. Methodology

A first step in the analysis was to quantify the seasonality of the main response variable (EDV) and the proposed predictors: maximum temperature and heat index. The aim was to compare the features of the seasonal cycles (peak time and amplitude) and their regional variability, as well as to assess the potential synchronicity among them.

A common way to quantify the time delay between two time series and their association strength is by calculating the sampling cross-correlation functions (CCF) and identifying the lag values where the highest sample cross-correlation occurs [10]. To minimize the effect of the autocorrelation of each time series on this calculation, we applied the STL decomposition (Seasonal–Trend decomposition using LOESS) to remove seasonal and non-seasonal trends in the data [11].

Finally, predictive models for EDV were fitted by incorporating the overall observed seasonal effect of the response, along with the effects of the predictors, which included either heat index or temperature. Regional variability in the strength of this dependence was accounted for by including additional random effects in the model. Sequential model comparison was conducted as additional predictors were added by evaluating model predictive accuracy using a training–testing data split, while model fit was assessed using the Bayesian Information Criterion (BIC).

### 3.1. Seasonality Quantification

Variables EDV, temperature and heat index are strongly seasonal across all HHS regions; therefore seasonality quantification using useful and data informed metrics becomes an important initial step to describe the temporal patterns of these variables. For EDV, temperature, and heat index, seasonality metrics were calculated following [12].

Let $Y_{tj}$ be the response variable (EDV) at time *t* for region *j*, and $X_{tj}$ be the temperature or heat index at time *t* and region *j*. A log transformation was applied to EDV since it is a count variable.

We initially model the response as a purely seasonal model with one harmonic:

(4) $$\begin{array}{cc} Y_{tj}= & \beta_{0j}+\beta_{sj}\sin\left(\phi t\right)+\beta_{cj}\cos\left(\phi t\right)+\varepsilon_{tj} \end{array}$$

where $\phi =\frac{2\pi }{365.25}$; $\beta_{0j}$, $\beta_{sj}$ and $\beta_{cj}$ are the unknown parameters for the one-harmonic periodic regression; and $\varepsilon_{jt}$ are random normal errors with zero mean and constant variance within region.

Model coefficients are estimated by region using maximum likelihood, giving us regression coefficient estimates, their standard errors and covariances for each region. From these estimates we calculate:

- Phase angle $\psi =\arctan(\beta_{sj}/\beta_{cj})$
- $\mathrm{Var}\left(\psi \right)=\frac{\beta_{cj}^{2}\;\sigma_{sj}^{2}\;+\;\beta_{sj}^{2}\;\sigma_{cj}^{2}\;-\;2\;\sigma_{\beta_{cj}\;\beta_{sj}}\;\beta_{sj}\;\beta_{cj}}{{\left(\beta_{cj}^{2}\;+\;\beta_{sj}^{2}\right)}^{2}}$
- Peak timing $P_{T}=\left\{\begin{array}{cc} \psi /\phi & \mathrm{if}\;\beta_{sj},\beta_{cj}>0\\ (\psi +\pi )/\phi & \mathrm{if}\;\beta_{cj}<0\\ (\psi +2\pi )/\phi & \mathrm{otherwise} \end{array}\right.$
- $\mathrm{Var}\left(P_{T}\right)=\frac{\sqrt{\mathrm{Var}(\psi )}}{\phi }$
- Amplitude $\gamma =\sqrt{\beta_{cj}^{2}+\beta_{sj}^{2}}$
- $\mathrm{Var}\left(\gamma \right)=\frac{\beta_{cj}^{2}\;\sigma_{sj}^{2}\;+\;\beta_{sj}^{2}\;\sigma_{cj}^{2}\;+\;2\;\sigma_{\beta_{cj}\;\beta_{sj}}\;\beta_{sj}\;\beta_{cj}}{\beta_{cj}^{2}\;+\;\beta_{sj}^{2}}$
- Intensity $\theta =e^{\gamma }$

These metrics are then summarized by regions and represented graphically to assess spatial variability within their geographical setting.

### 3.2. Emergency Department Visits (EDV) Modeling

Enhancing effects of extreme air temperature and heat index are expected to produce amplified responses in the corresponding health-related outcomes. Under these assumptions, EDV may be subject to an overall seasonal effect that can be modified by localized seasonal effects, as well as by the impacts of temperature or heat index, which may also vary across regions due to local conditions.

Two sets of models are proposed to represent EDV outcomes, using either temperature or heat index as potential predictors. In total, six generalized linear mixed-effects models (GLMMs) were considered. Let $X_{tj}$ denote the temperature or heat index at time *t* for region *j*, and let $\mu_{tj}=E\left(Y_{tj}\right)$ denote the expected value of EDV. The link function is denoted by g(·).

In these models, parameters $b_{s}$ and $b_{c}$ are the corresponding fixed effects regression coefficients for the one-harmonic sine and cosine functions, while $\beta_{sj}$ and $\beta_{cj}$ are corresponding random effects with values changing across regions. $\beta_{x}$ is the fixed effect regression coefficient for the external climatic predictors (maximum temperature or heat index) while $\beta_{xj}$ is the corresponding random effect with values changing across regions. Parameter $\beta_{0j}$ represents a random intercept or baseline value which changes across regions. The random effects are assumed to follow mean-zero distributions with variance components to be estimated from the data.

Let $X_{tj}$ be the temperature or heat index at time *t* for region *j*. The models are:

1:  $g\left(\mu_{tj}\right)=b_{s}\sin\left(\phi t\right)+b_{c}\cos\left(\phi t\right)+\beta_{0j}$
2:  $g\left(\mu_{tj}\right)=b_{s}\sin\left(\phi t\right)+b_{c}\cos\left(\phi t\right)+\beta_{sj}\sin\left(\phi t\right)+\beta_{cj}\cos\left(\phi t\right)+\beta_{0j}$
3:  $g\left(\mu_{tj}\right)=b_{s}\sin\left(\phi t\right)+b_{c}\cos\left(\phi t\right)+\beta_{0j}+b_{x}X_{tj}$
4:  $g\left(\mu_{tj}\right)=b_{s}\sin\left(\phi t\right)+b_{c}\cos\left(\phi t\right)+\beta_{0j}+b_{x}X_{tj}+\beta_{xj}X_{tj}$
5:  $g\left(\mu_{tj}\right)=b_{s}\sin\left(\phi t\right)+b_{c}\cos\left(\phi t\right)+\beta_{sj}\sin\left(\phi t\right)+\beta_{cj}\cos\left(\phi t\right)+\beta_{0j}+b_{x}X_{tj}$
6:  $g\left(\mu_{tj}\right)=b_{s}\sin\left(\phi t\right)+b_{c}\cos\left(\phi t\right)+\beta_{sj}\sin\left(\phi t\right)+\beta_{cj}\cos\left(\phi t\right)+\beta_{0j}+b_{x}X_{tj}+\beta_{xj}X_{tj}$

where g(.) is the link function and $\mu_{tj}=E\left[EDV_{tj}\right]$ is the expected value of EDV for time *t* and region *j*.

Models 1 and 2 exclude the effect of an environmental predictor and are purely seasonal, while the remaining models 4 to 6 include the effect of an environmental variable (maximum temperature or heat index) on the EDV response. Models 2, 5 and 6 include both a fixed global seasonal effect and a random seasonal effect that varies across regions. Models 4 and 6 include both a fixed global effect of *X* over EDV, and a random effect of *X* over EDV that changes across regions. While model 1 considers a random intercept which modifies the baseline values of EDV from region to region, Model 2 also accounts for regional modifications of the seasonal effects over the global seasonal signal, by adding random effects on the sine and cosine components.

### 3.3. Model Diagnostics

All models were compared in terms of predictability, by estimating the model parameters using a training set, and calculating the mean squared error (MSE) on a testing set, and in terms of goodness of fit. Different train-test split data percentages were compared to evaluate the sensitivity of the prediction errors to the different split schemes.

Temperature models were evaluated using both the full data (to the end of 2025) and data limited to the heat index time range (until the end of 2022). To test the sensitivity of different train-test data splits, different percentages were considered for each data range.

Possible overdispersion was accounted for by using the Negative Binomial 2 family as the probability distribution for the response. Model comparisons between the Poisson and Negative Binomial 2 (NB2) families were conducted. The Bayesian Information Criterion (BIC) was used to evaluate how well a model captures the overall distribution of the observed EDV counts when used within the context of model comparison.

Vulnerability variables were initially evaluated as potential predictors in the temperature and heat index models. These variables were aggregated to the HHS regional level using population-weighted averages. Because the vulnerability measures were available only at the annual scale, each annual estimate was assigned to all daily observations within the corresponding year to align with the daily EDV time series. The vulnerability predictors did not improve model performance in terms of Mean Square Error, and model comparisons based on the Bayesian Information Criterion (BIC) indicated that their inclusion did not improve model fit. Consequently, these variables were not retained in the final analyses.

The intra-class correlation coefficient (ICC), also referred to as the variance partition coefficient, measures the proportion of total variability attributable to the random effects associated with the data clusters (i.e., region-to-region variability). Fixed effects influence the ICC indirectly by reducing the unexplained variance components used in its calculation. To quantify the contribution of regional variability in emergency department visits (EDV), including seasonal components and covariate effects, we computed ICC values both before adjusting for model covariates (unadjusted ICC) and after including covariates such as seasonal effects and temperature or heat index (adjusted ICC).

Residual analysis is a critical step in regression model diagnostics, used to detect model misspecification, including incorrect functional forms, distributional assumptions, or the presence of influential outliers. For generalized linear mixed models (GLMMs) with non-normal responses, a simulation-based residual approach is recommended over traditional Pearson and deviance residuals [13]. In this framework, residuals are defined as simulation-based quantile residuals taking values in the interval [0,1]. They represent the position of each observed value within the cumulative distribution of responses simulated from the fitted model, i.e., $r_{i}=F_{model}\left(y_{i}\right)$. This simulation-based quantile residual approach, implemented in the R package DHARMa [14], was used for residual diagnostics.

## 4. Results

The seasonality analysis of the main time series in this research is presented in Section 4.1. As an initial step, the time series are treated as independent across the different HHS regions, and separate models are fitted for each region. Exploration about the delay between the environmental time series and the EDV response time series is presented in Section 4.2. Following this analysis, we present the EDV predictive models that include maximum temperature as the main external covariate in Section 4.3 (maximum temperature models), followed by the EDV predictive models that use the heat index as an external covariate in Section 4.4 (heat index models).

### 4.1. Seasonality Analysis

**Figure 2:**
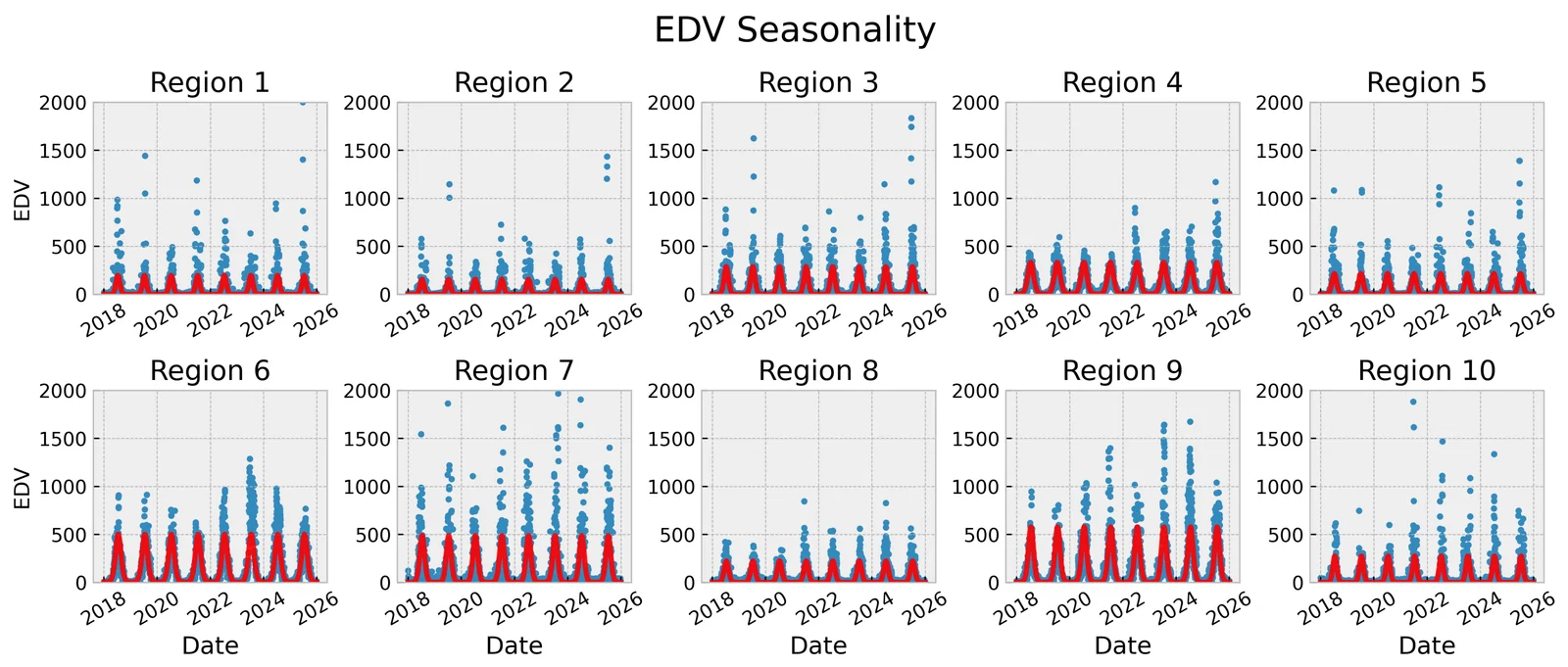
Seasonal models for EDV (per 100,000 population).

**Figure 3:**
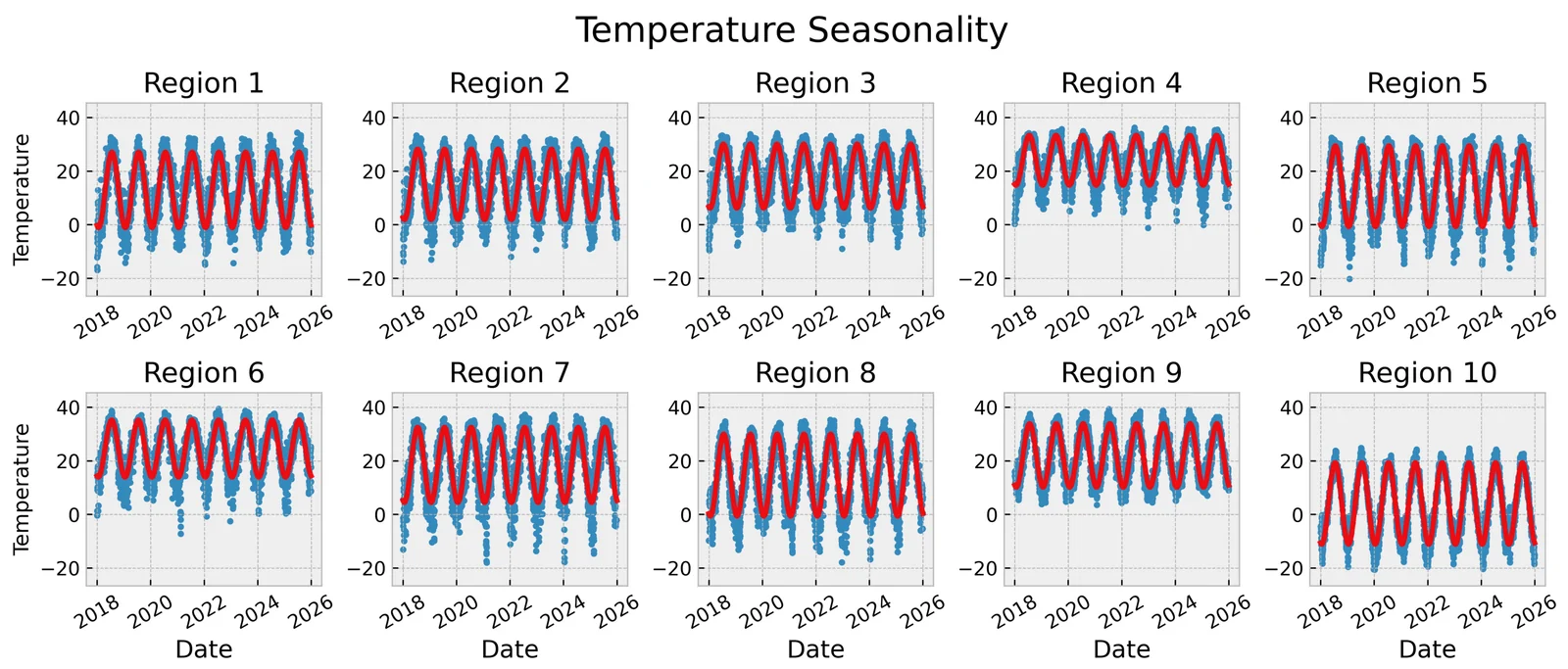
Seasonal models for temperature (degrees Celsius).

**Figure 4:**
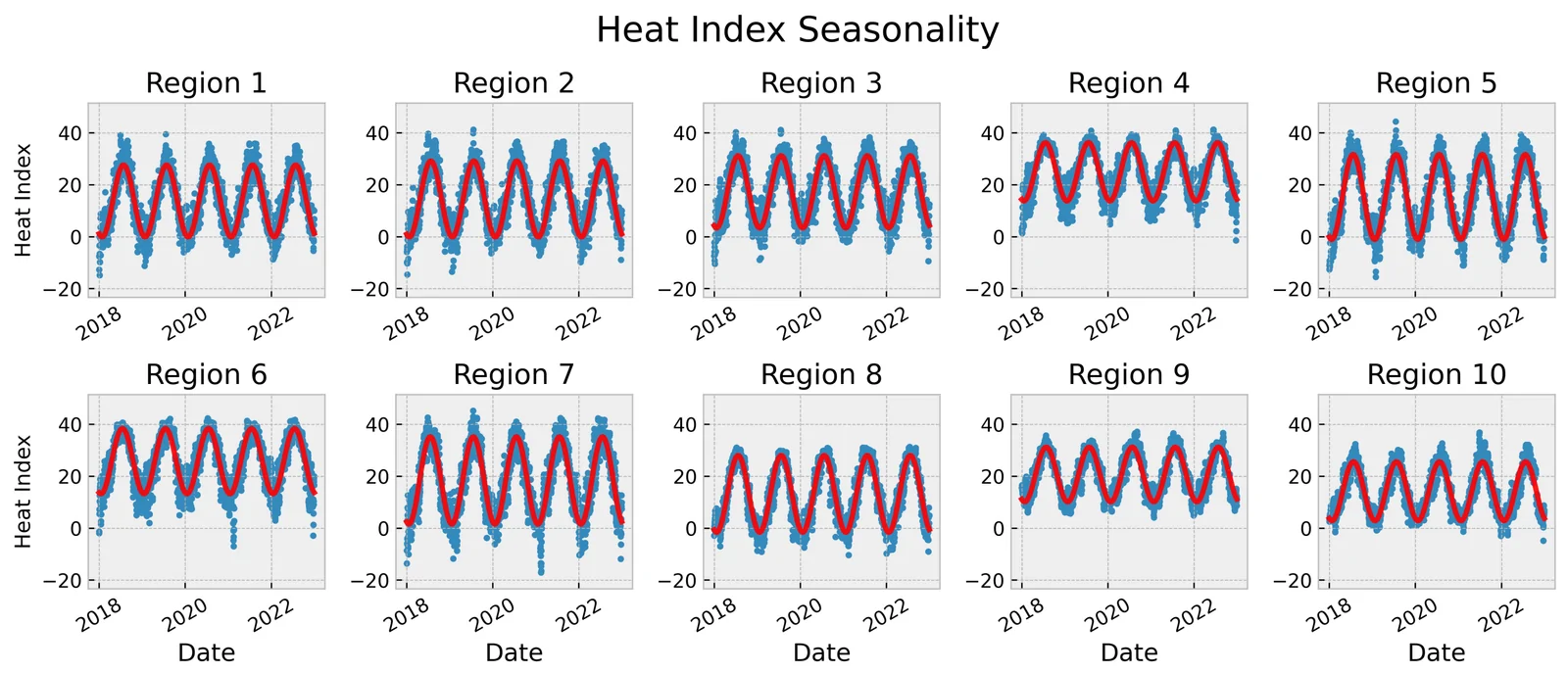
Seasonal models for heat index (degrees Celsius).

The one-harmonic seasonal models fitted to the observed time series of EDV, maximum temperature and heat index are shown in Figure 2, Figure 3, and Figure 4, respectively. The model (red lines) is able to reproduce the overall seasonal behavior for all time series at each HHS region, more specifically, the peak timing, but they are not able to reproduce the more extreme events (blue dots). Representation of regional differences in amplitude and inter-annual variability requires additional model components or covariates.

Seasonality metrics for EDV, temperature, and heat index are shown in Table A1, Table A2, and Table A3, respectively. These results show an average peak timing across regions of 194, 200.4 and 203.4 days from the beginning of the year for EDV, maximum temperature and heat index respectively. The EDV peak occurs earlier than the peak timings for both maximum temperature and heat index. There is a difference of nearly one week between the average peak timing of EDV and that of maximum temperature. This difference increases to nearly 10 days on average when comparing the average peak timing of EDV with that of the heat index.

**Figure 5:**
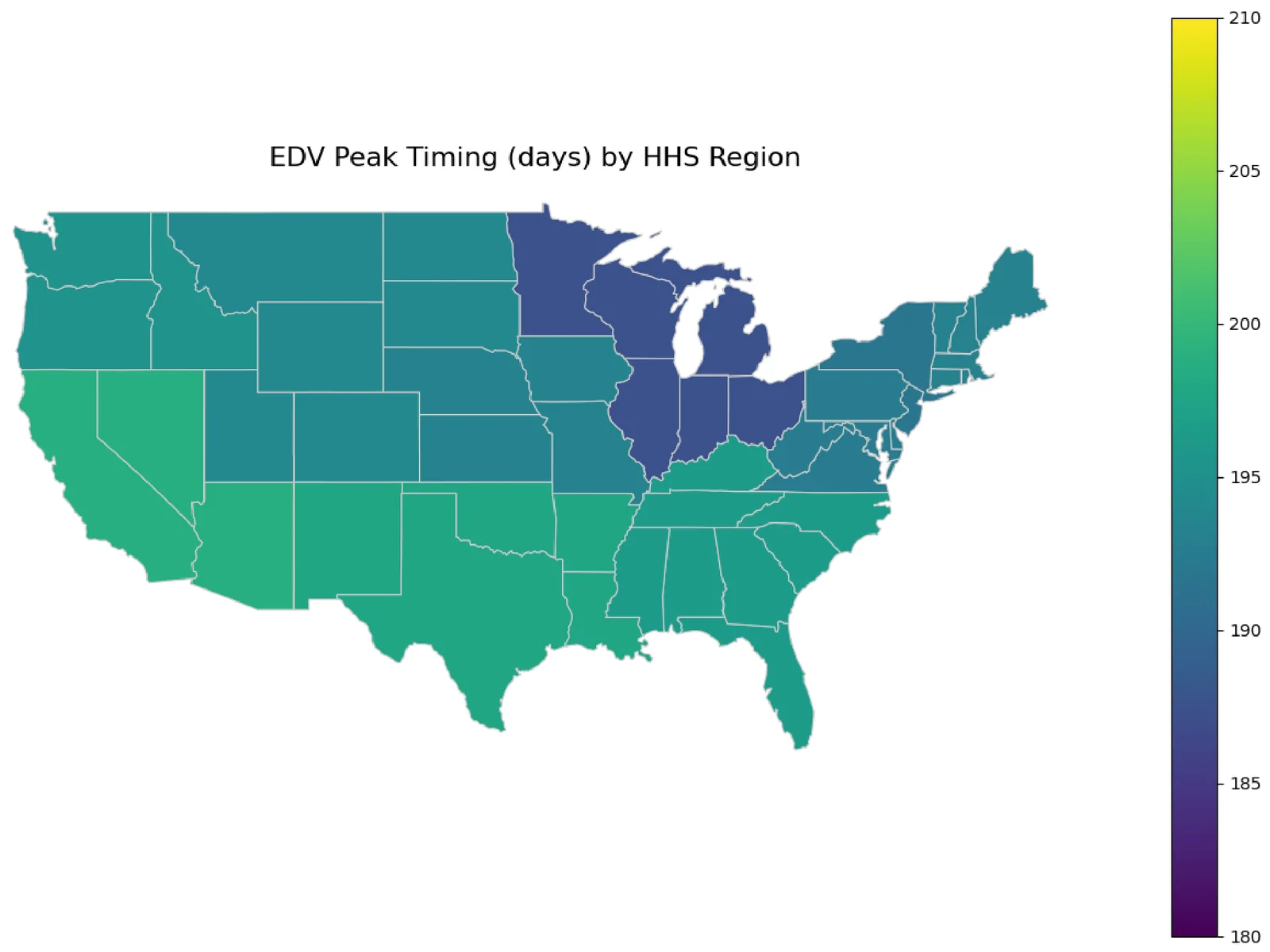
Peak Timing for EDV (days from the beginning of the year).

**Figure 6:**
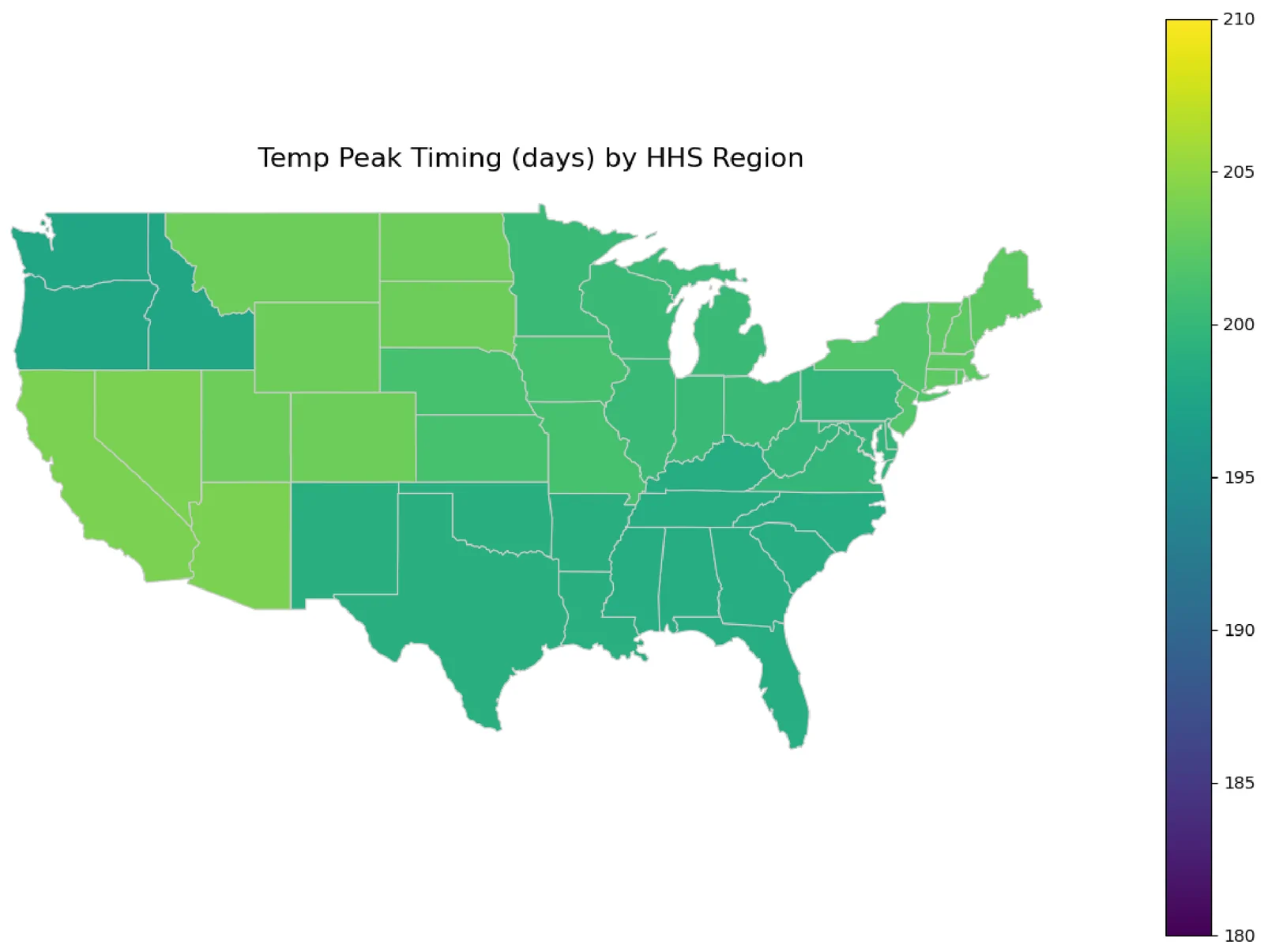
Peak Timing for Maximum Temperature (days from the beginning of the year).

**Figure 7:**
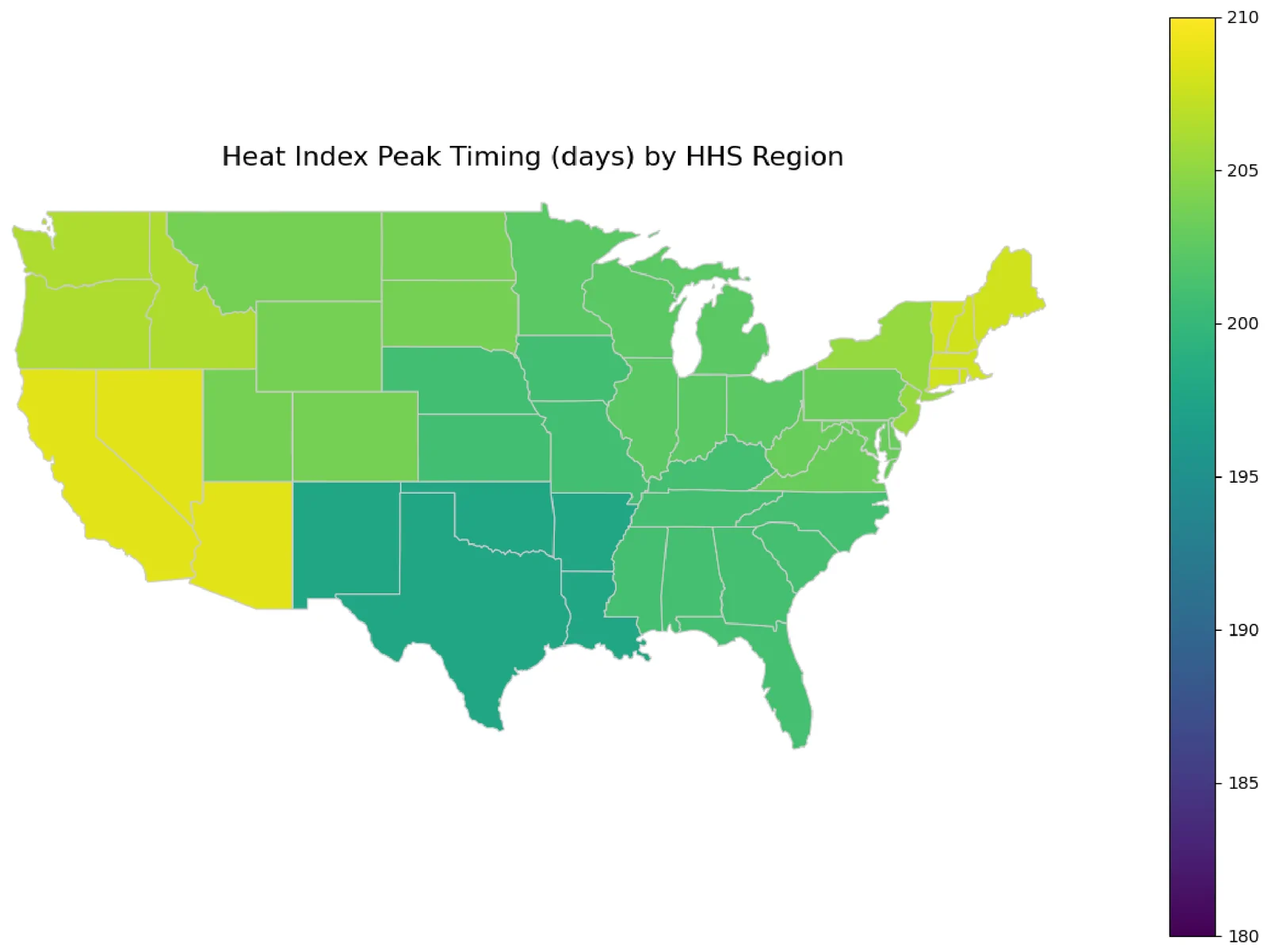
Peak Timing for Heat Index (days from the beginning of the year).

Figure 5, Figure 6, and Figure 7 show maps of the estimated peak times for EDV, temperature, and heat index, respectively. The earliest peak timing for EDV occurs in HHS Region 5 (Illinois, Indiana, Michigan, Minnesota, Ohio, and Wisconsin), while the latest peak occurs in Region 9 (Arizona, California, and Nevada) among the continental states. This pattern is not necessarily synchronized with the earliest and latest peak times for maximum temperature and heat index.

An inspection of detailed regional differences between the response and the seasonal effects reveals that a maximum difference of two weeks occurs in Region 5 (Illinois, Indiana, Michigan, Minnesota, Ohio, Wisconsin) when comparing EDV and heat index peak timing, while a minimum difference of one-quarter of a day between peak times occurs in Region 6 (Arkansas, Louisiana, New Mexico, Oklahoma, Texas). Similar extreme differences in peak timings are observed between EDV and maximum temperature for the same regions. These regional differences are accounted for through random effects that represent the region-to-region variability of the seasonal cycle model representation.

### 4.2. Lag Detection Between EDV and Environmental Predictor Variables

**Figure A1:**
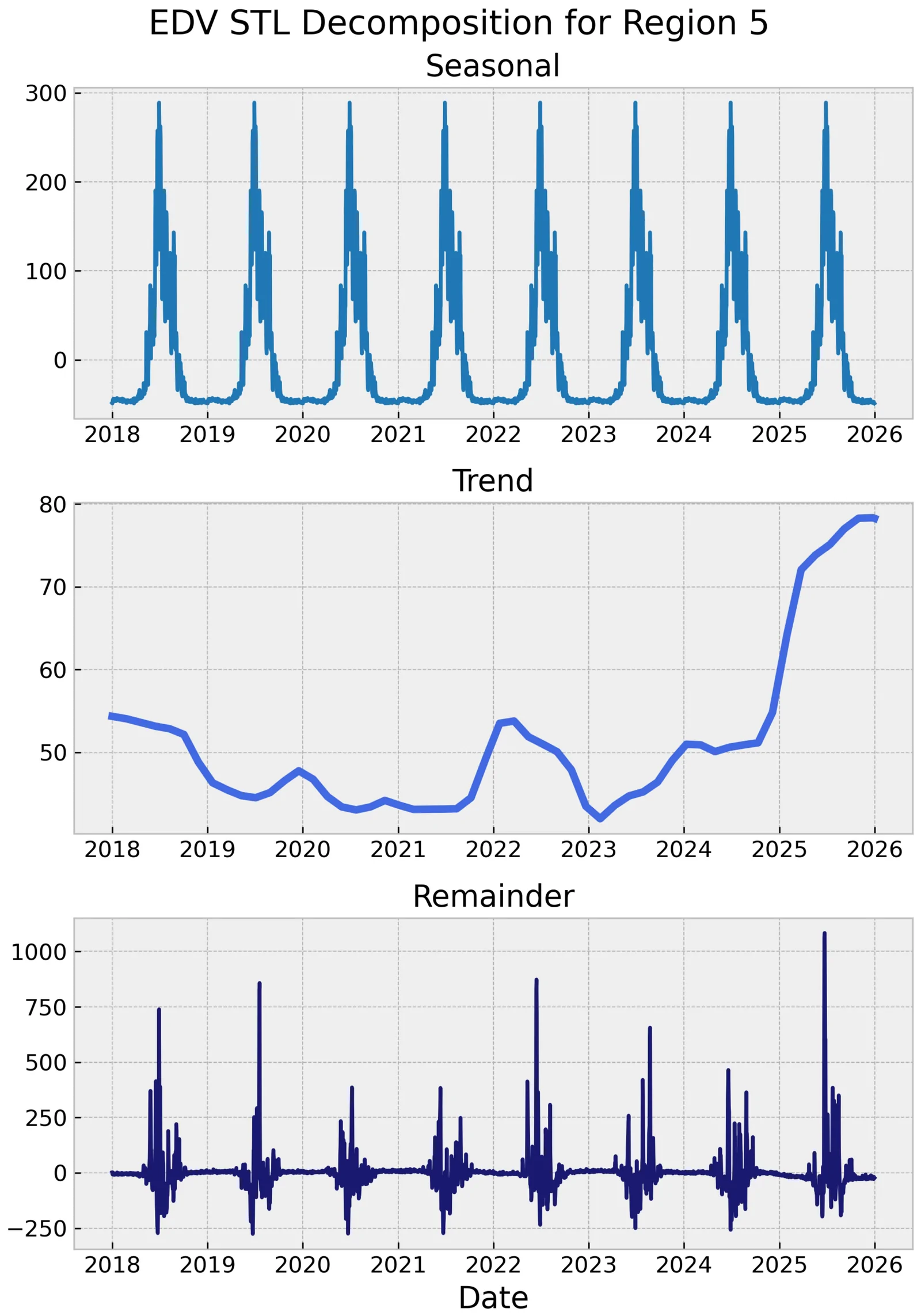
STL decomposition for EDV region 5.

**Figure A2:**
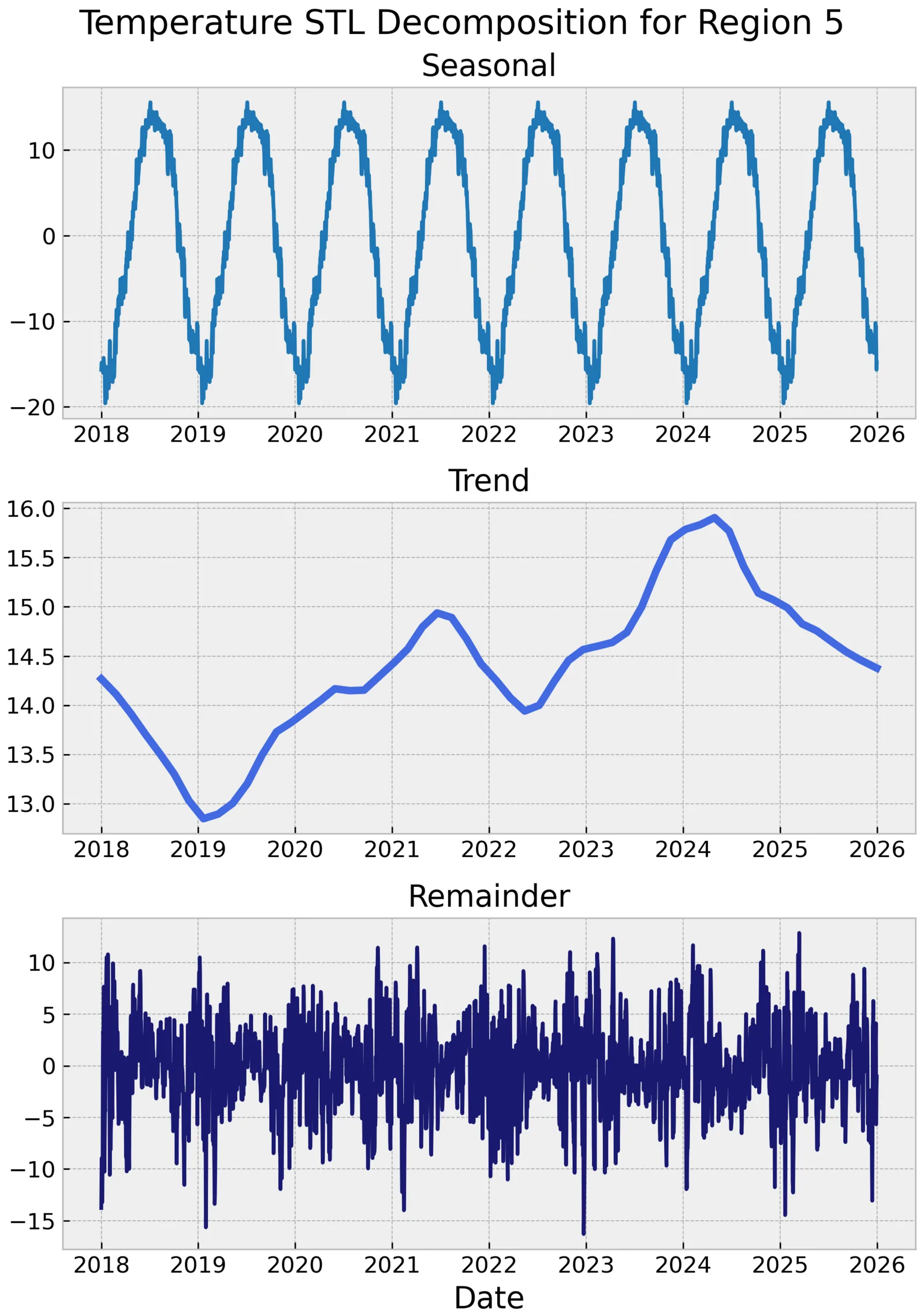
STL decomposition for temperature region 5.

**Figure A3:**
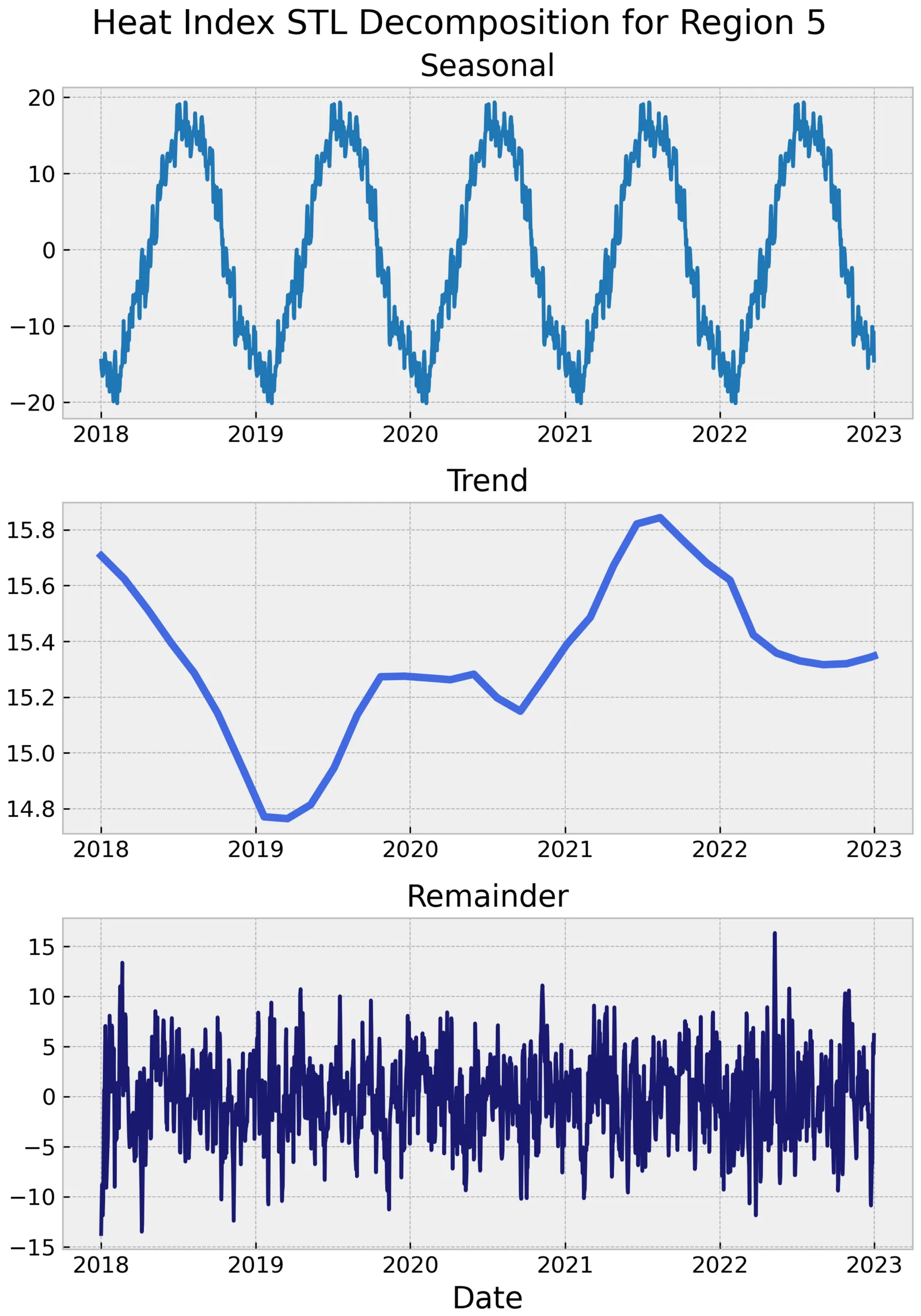
STL decomposition for heat index region 5.

STL decompositions for EDV, temperature, and heat index are shown in Figure A1, Figure A2, and Figure A3, respectively.

**Figure A4:**
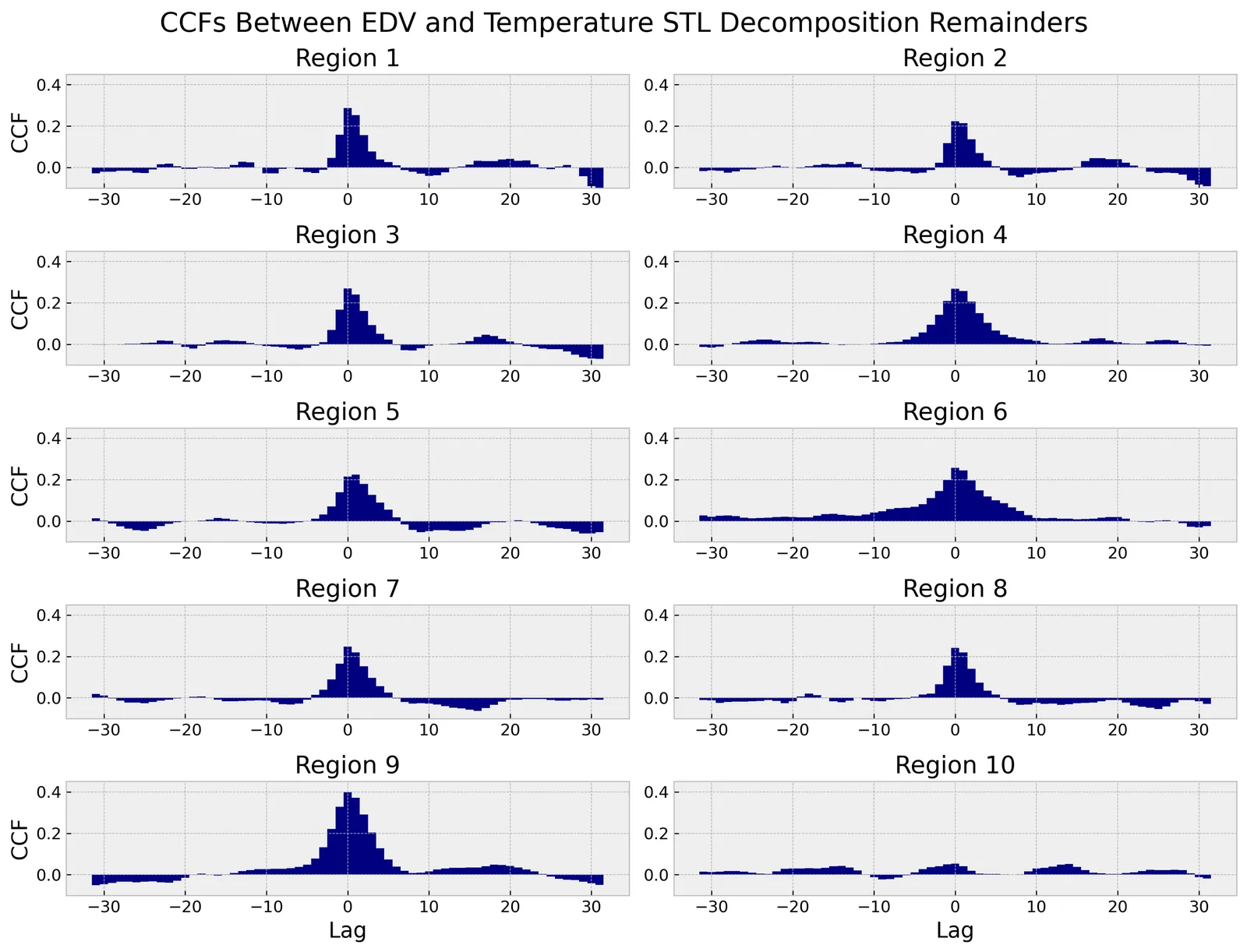
Cross-correlations between EDV and temperature STL remainders.

**Figure A5:**
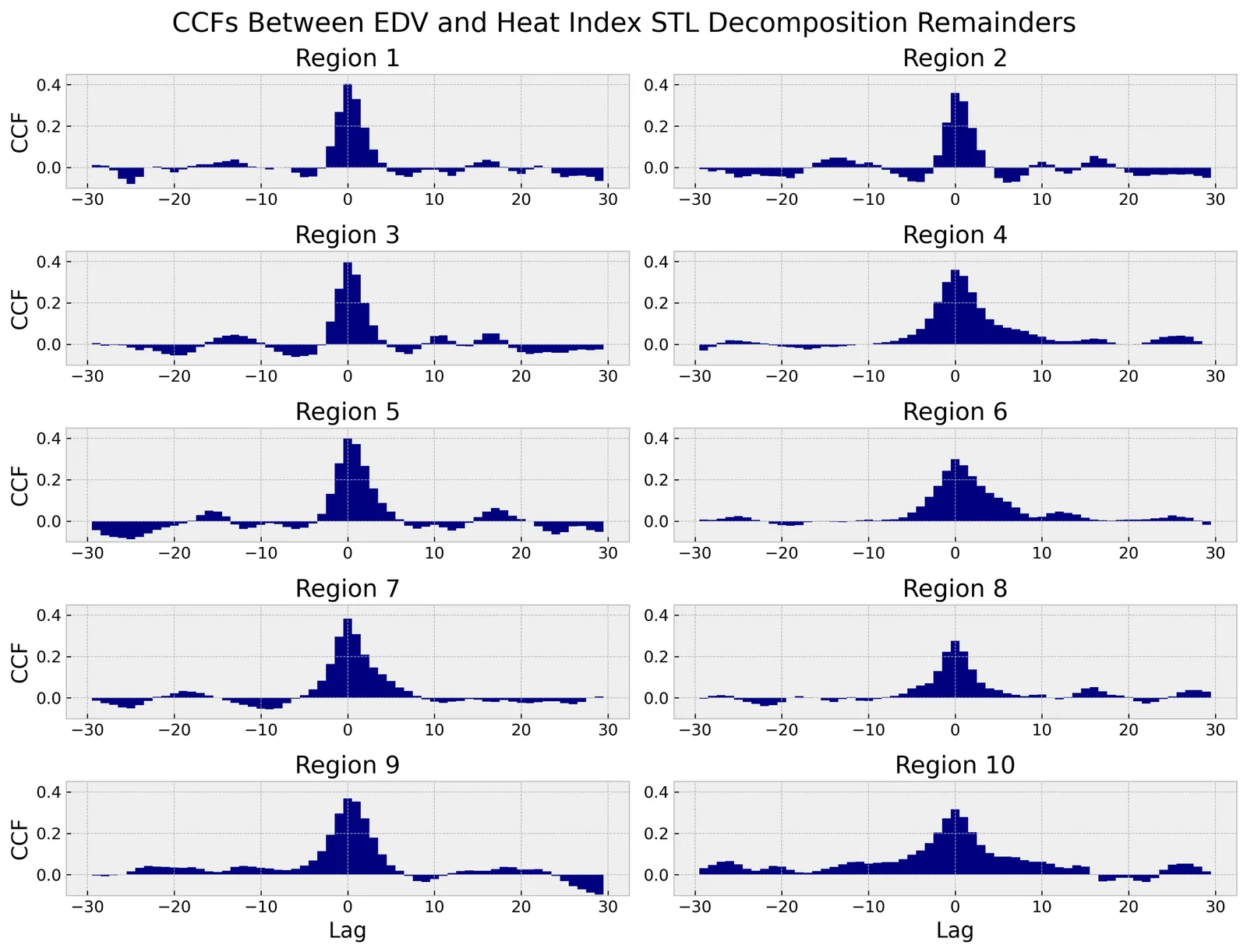
Cross-correlations between EDV and heat index STL remainders.

Cross-correlations between the STL remainders for EDV and temperature and EDV and heat index are shown in Figure A4 and Figure A5, respectively.

In most cases, except for the CCF between EDV and maximum temperature residuals in Region 5, the maximum cross-correlation values were generally observed at lag zero, suggesting that the strongest detectable association between environmental conditions and EDV occurs contemporaneously at the temporal and spatial scales examined in this study. In view of these results, lagged predictors were not included in the proposed models described in the following section. However, this finding should not be interpreted as evidence that delayed heat-health effects are absent.

### 4.3. Temperature Models

**Figure 8:**
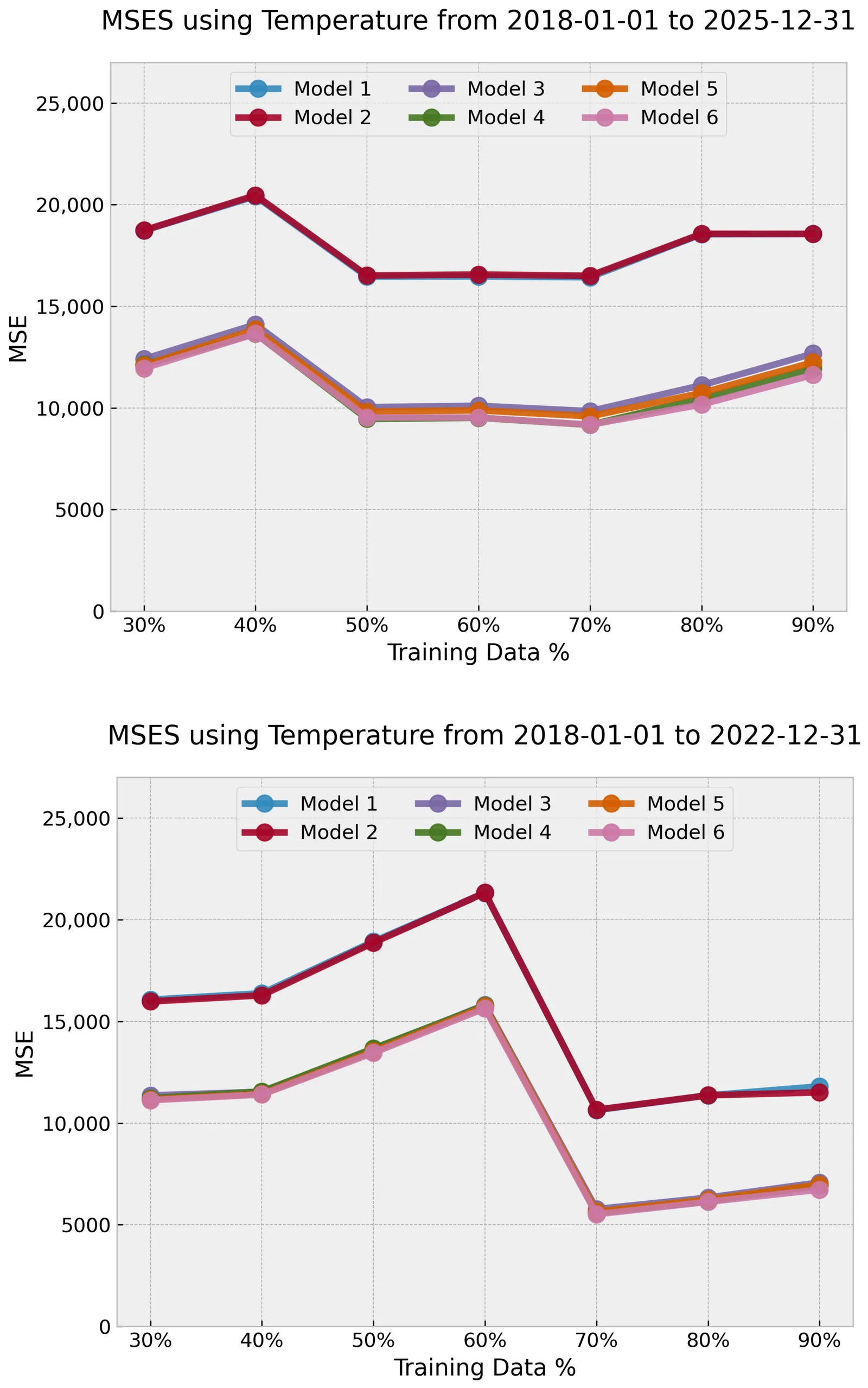
MSEs for temperature models at different train-test splits. **Top**: Data ending in 2025. **Bottom**: Data ending in 2022.

Figure 8 compares the MSEs for models 1 to 6, including temperature as a predictor in models 3 to 6.

As shown in Figure 8, models 3, 4, 5, and 6 had much lower MSEs than models 1 and 2. The lowest MSEs occurred at a 70-30 train-test split for both data ranges, with model 4 and model 6 being optimal for the data ending in 2025 and 2022, respectively. However, the difference in MSEs was very small, and all four models including temperature had a similar performance.

**Figure 9:**
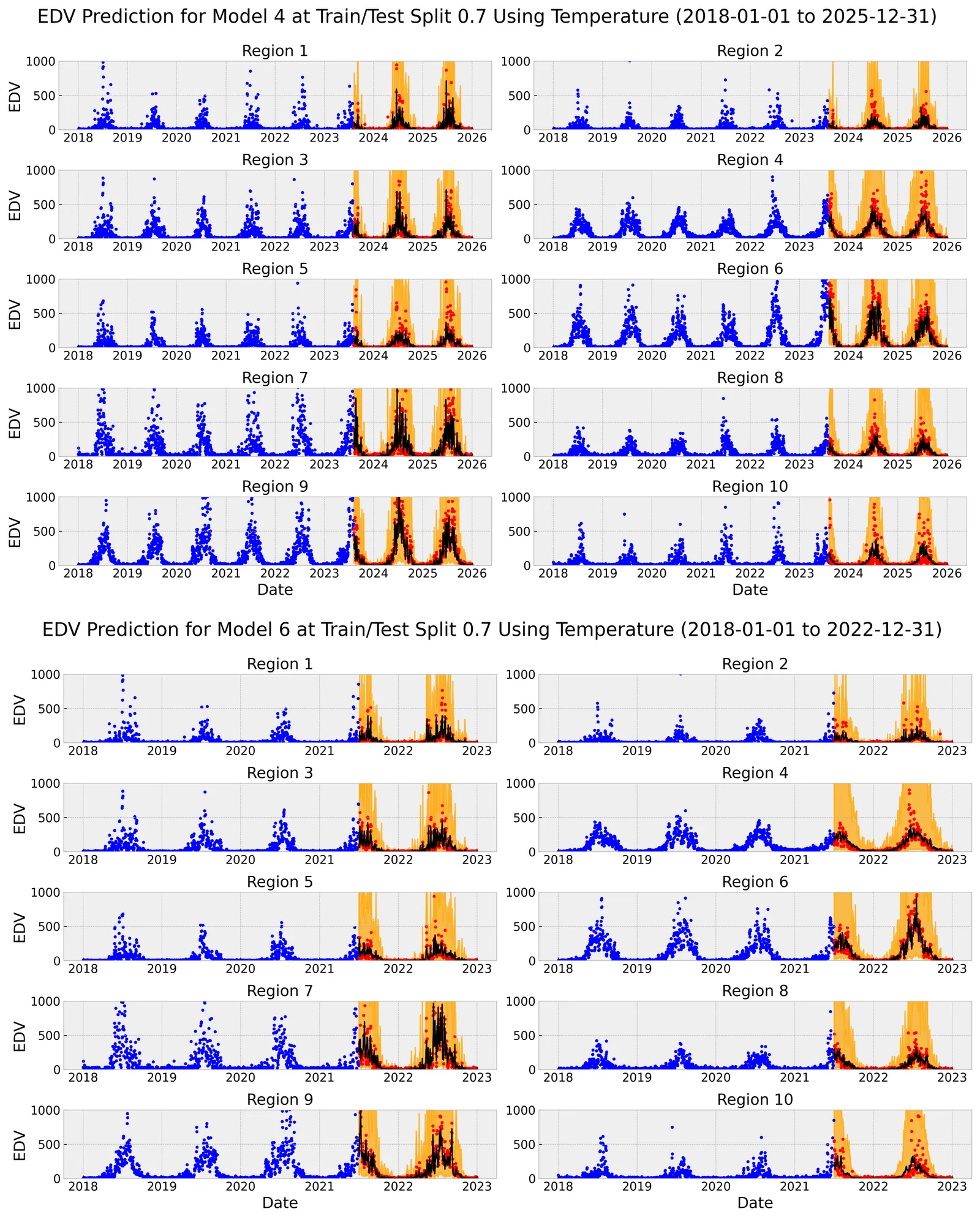
Temperature model predictions using a 70-30 train-test split. Training data shown in blue and test data shown in red. The black line represents the model predictions and the orange lines represent the approximate 95% predictions intervals (±2 standard errors). **Top**: Data ending in 2025 using model 4. **Bottom**: Data ending in 2022 using model 6.

The best two models, models 4 and 6, were used to make predictions in the testing set. Figure 9 shows the predictions for these two models for all HHS regions. Both models suggest distinct regional differences in the associations between EDV and maximum temperature.

### 4.4. Heat Index Models

**Figure 10:**
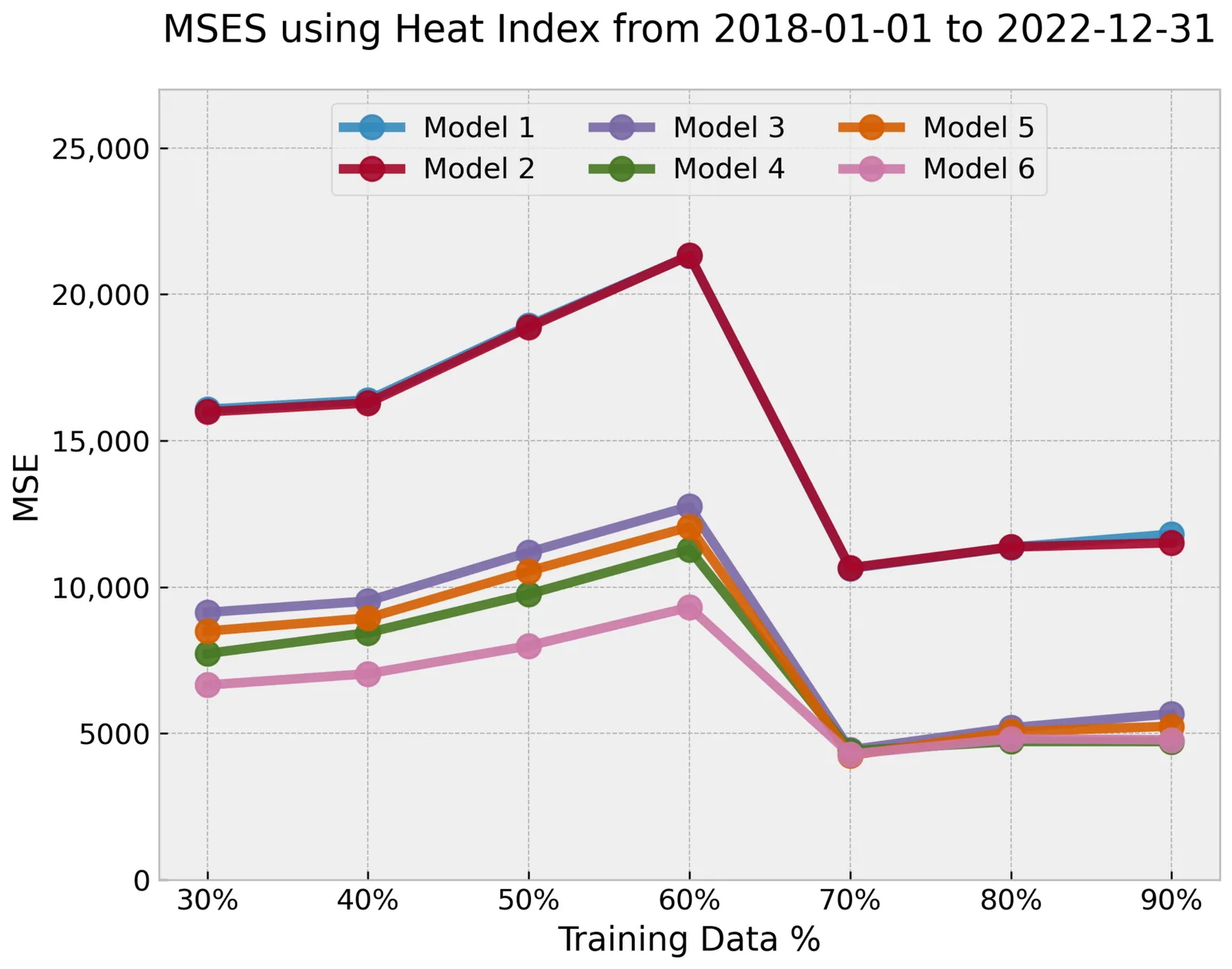
MSEs for heat index at different train-test splits. Data ends in 2022.

Heat index models were evaluated using data that ends at the end of 2022, as shown in Figure 10. Similar to the temperature models, models 3, 4, 5, and 6 had much lower MSEs than models 1 and 2 (which did not include heat index as a predictor). Though the four models with heat index show similar predictive performance at a 70-30 train-test split, model 5 had the lowest MSE.

**Figure 11:**
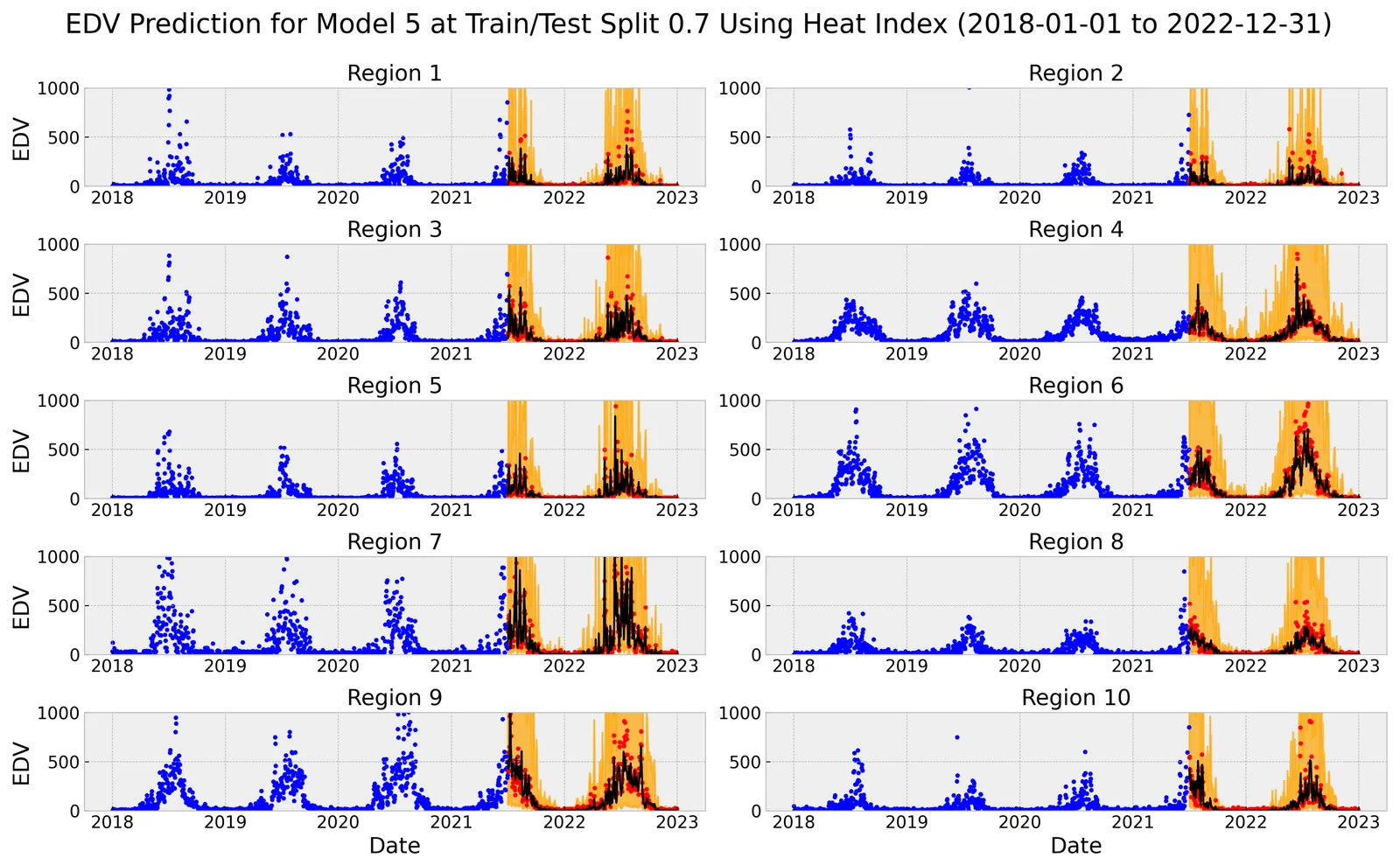
Predictions for heat index models with the optimal model (model 5) and a 70-30 train-test split. Training data shown in blue and test data shown in red. The black line represents the model predictions and the orange lines represent the approximate 95% predictions intervals (±2 standard errors).

Figure 11 shows the predictions for the model with the best predictive performance (model 5). Results are shown for all HHS regions.

In contrast with the best predictive models selected in the temperature case, model 5 suggests a more homogeneous dependence of EDV on the environmental predictor (heat index) across regions, excluding a random effect for the dependence between the mean EDV and the heat index. An estimated regression coefficient ${\hat{b}}_{x}=0.08924$ implies an overall 9% increase in the mean EDV rate per unit increase in the heat index.

Both temperature and heat index models are able to adequately capture the seasonal patterns of EDV. Extreme observations generally lie within the 95% prediction intervals but remain more difficult to reproduce, possibly due to the presence of overdispersion, which is not accounted for by the Poisson model.

### 4.5. Fitting Performance vs. Prediction Accuracy for Temperature and Heat Index Models

**Figure 12:**
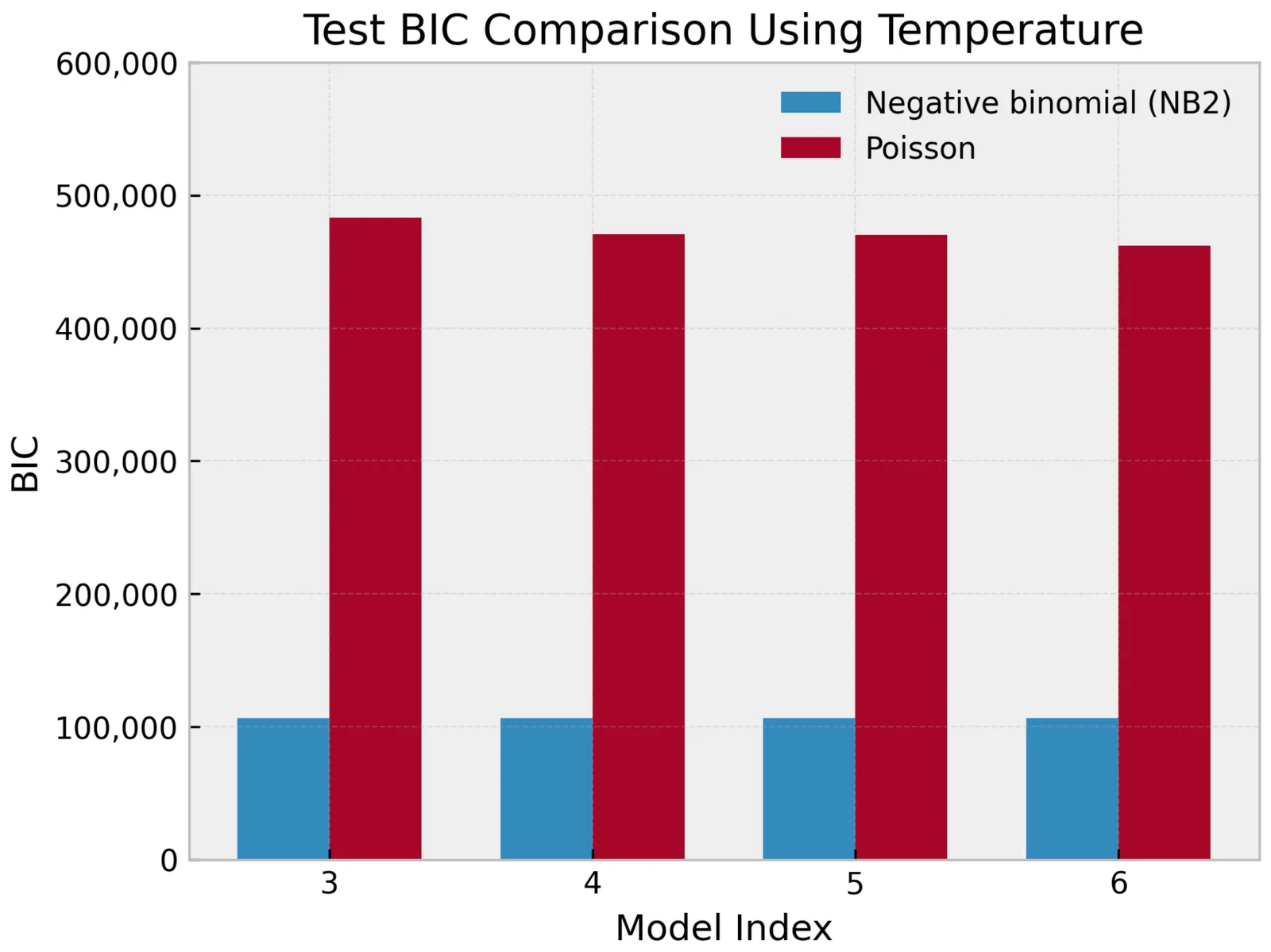
Temperature model comparisons using the Bayesian Information Criteria (BIC). Lower values indicate better model fit.

**Figure 13:**
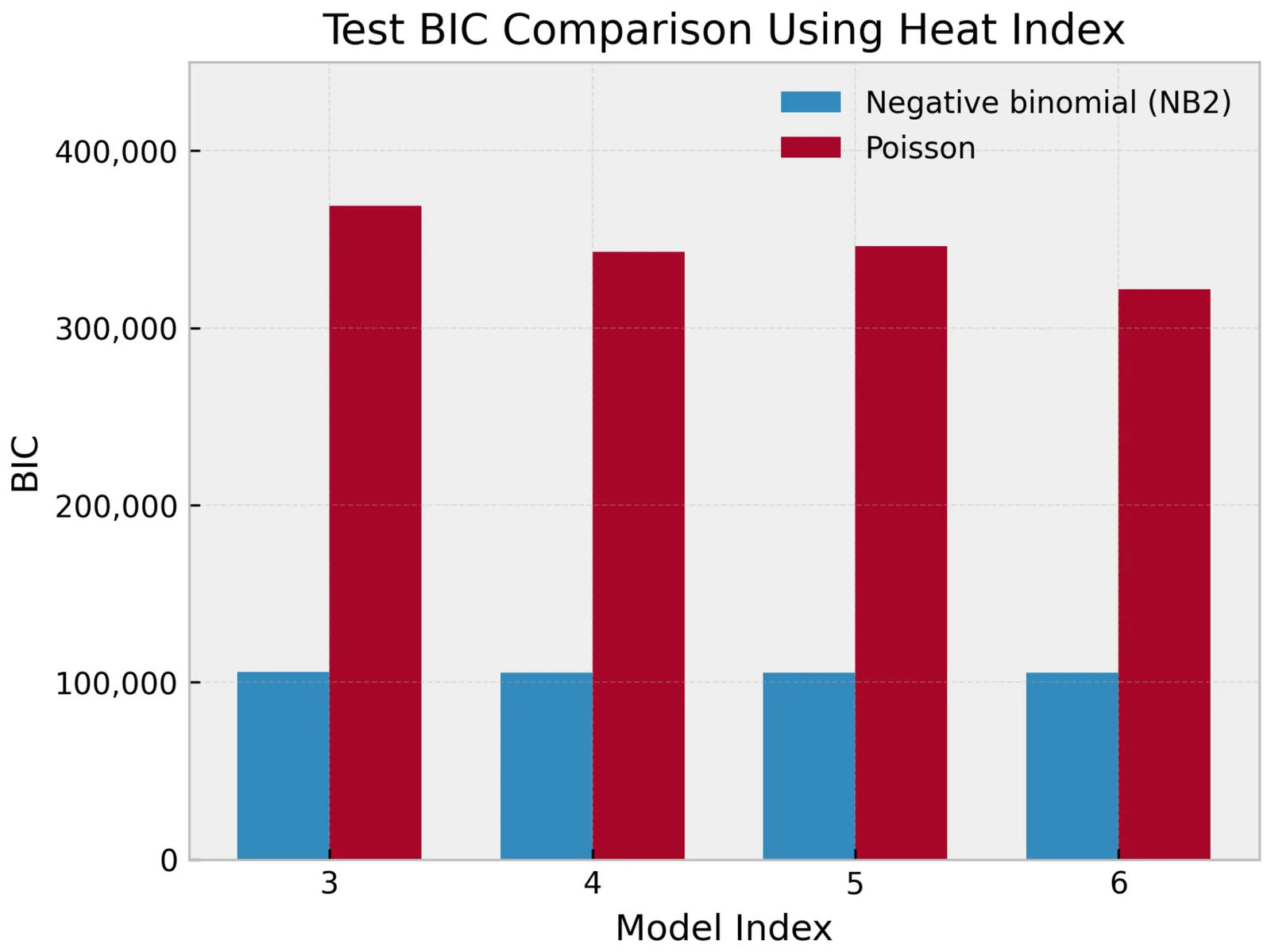
Heat Index model comparisons using the Bayesian Information Criteria (BIC). Lower values indicate better model fit.

Lower values of BIC indicate better goodness of fit after penalizing for model complexity. In this case, the data exhibit clear overdispersion, meaning that the variance exceeds the mean. The Poisson model imposes equality between the mean and variance, which leads to an inadequate representation of the variability in the data, particularly in the tails. In contrast, the NB2 model relaxes this restriction by allowing the variance to exceed the mean, enabling it to better capture the observed dispersion. As a result, the NB2 model provides a substantially higher likelihood by fitting the full distribution more accurately. This improvement in likelihood outweighs the modest complexity penalty in BIC, yielding lower (better) BIC values as shown in Figure 12 and Figure 13 for temperature and heat index models respectively.

**Figure 14:**
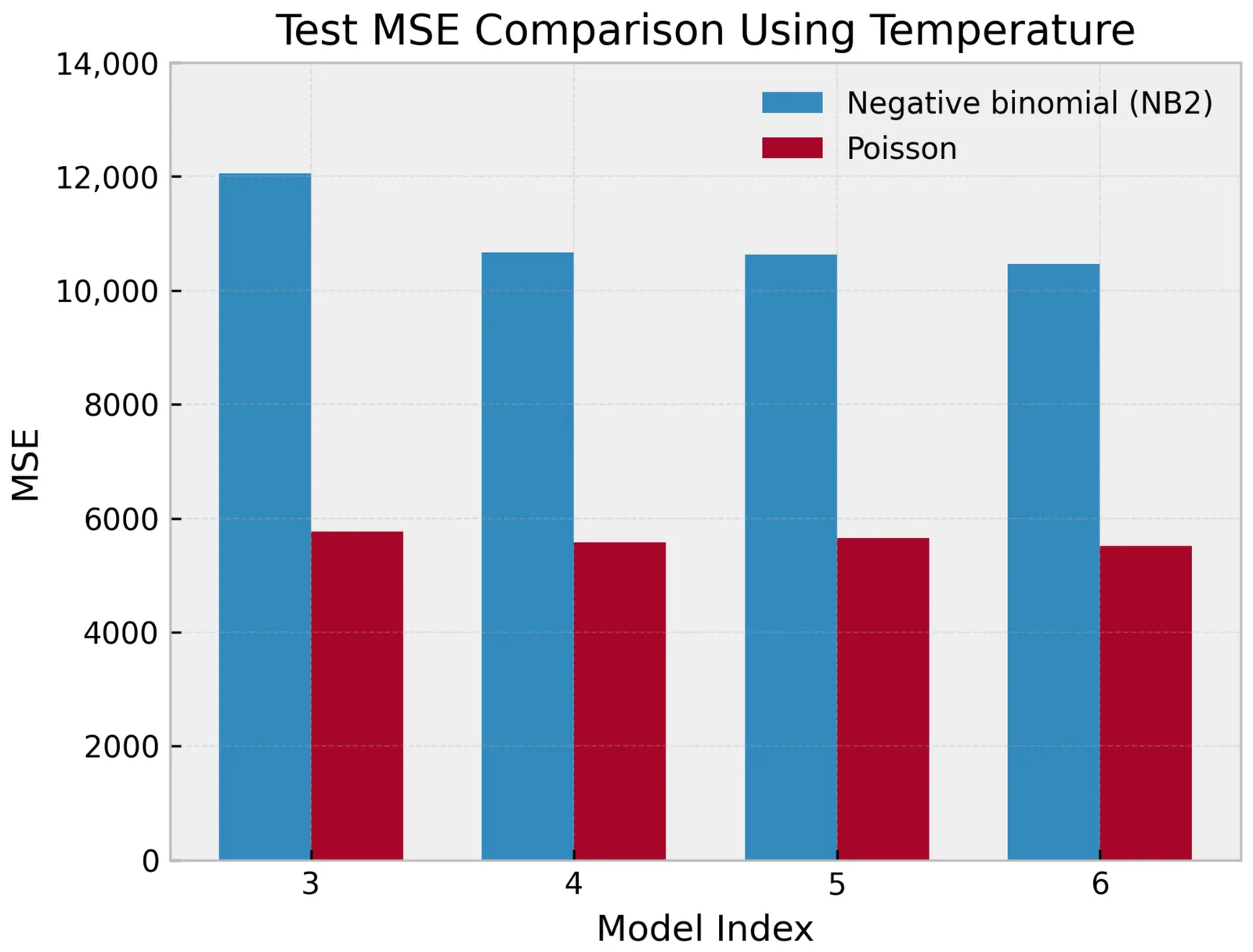
Temperature model predictive power measured by Mean Square Error (MSE) using the data period. Lower values indicate better predictive performance.

**Figure 15:**
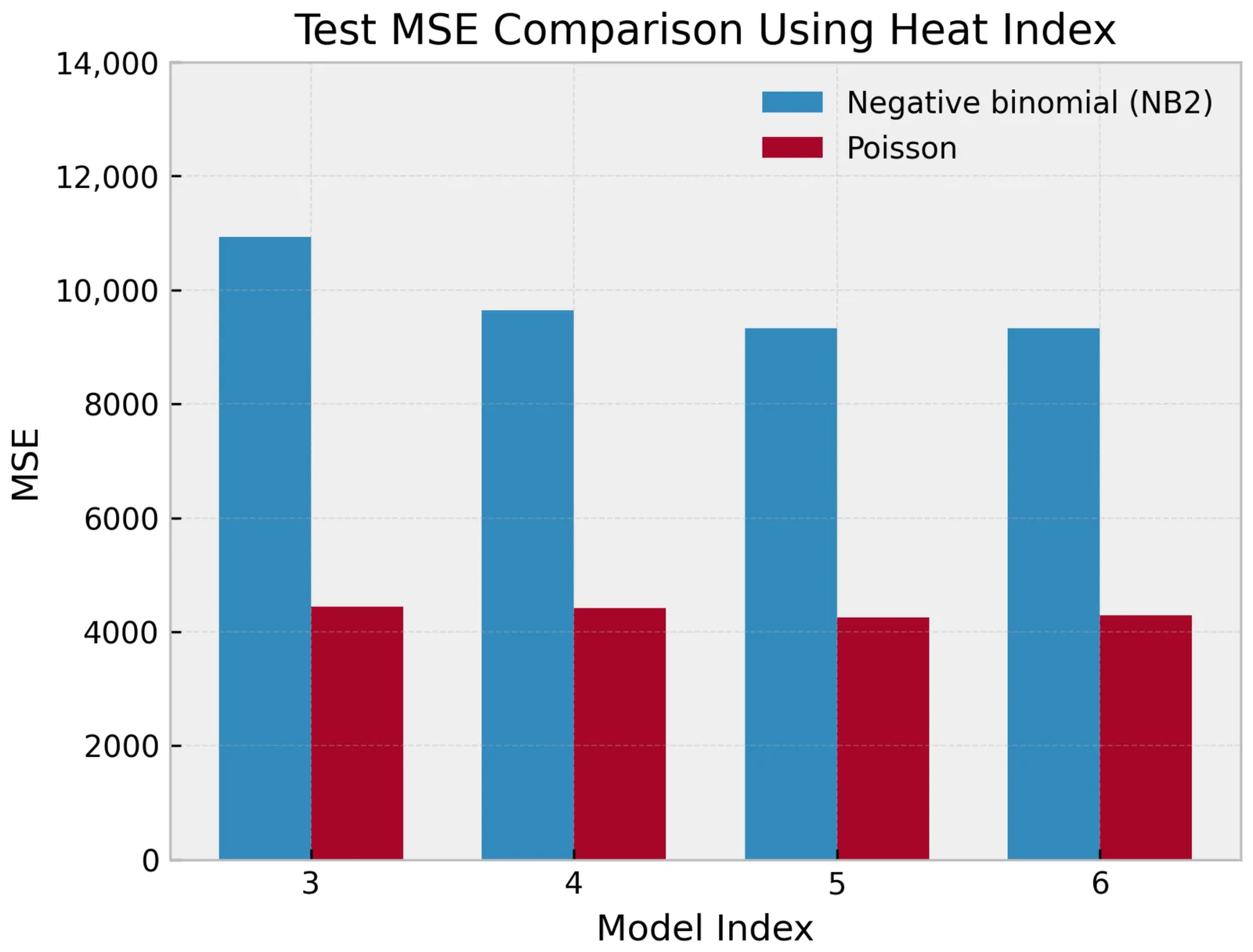
Heat Index model predictive power measured by Mean Square Error (MSE). Lower values indicate better predictive performance.

The Poisson model can achieve better predictive performance in terms of MSE, despite providing an inferior fit to the overall distribution. Figure 14 and Figure 15 show a comparison of the MSEs between the Poisson family and Negative Binomial 2 family for the temperature and heat index models respectively.

Although the Negative Binomial model provided a better fit to the observed count distribution, the primary goal of this study was predictive accuracy rather than parameter inference. Across repeated cross-validation analyses, the Poisson model consistently achieved lower prediction error (MSE), indicating superior out-of-sample predictive performance. Consequently, model selection was based primarily on predictive utility, while the Negative Binomial results were retained as the primary inferential model. We emphasize that the choice of the Poisson model as a predictive benchmark, does not imply that overdispersion is absent, but rather reflects its stronger predictive performance for the forecasting objective considered here. The superior predictive performance of the Poisson model does not imply that it necessarily provides the most reliable parameter estimates, standard errors, or uncertainty quantification. For inferential purposes, the Negative Binomial model remains an appropriate alternative because of its ability to accommodate extra-Poisson variability.

### 4.6. Intra-Class Correlation Coefficient

Table 1 presents the ICC estimates for the best-performing temperature and heat index models under both the Poisson and Negative Binomial type 2 (NB2) families. For the Poisson models, unadjusted ICC values are relatively low (approximately 5–10%), indicating modest baseline clustering and that only a small fraction of the total variability is attributable to differences between regions prior to covariate adjustment.

For the Negative Binomial models, adjusted ICC values remain substantially lower (approximately 0.10–0.14) than those from the Poisson models. This reflects the additional observation-level variability introduced by the NB2 dispersion parameter, which accounts for overdispersion beyond the Poisson assumption. The increased within-region heterogeneity inflates the total variance, thereby reducing the proportion attributable to between-region variation and leading to smaller ICC values even after covariate adjustment.

### 4.7. Residual Analysis

**Figure 16:**
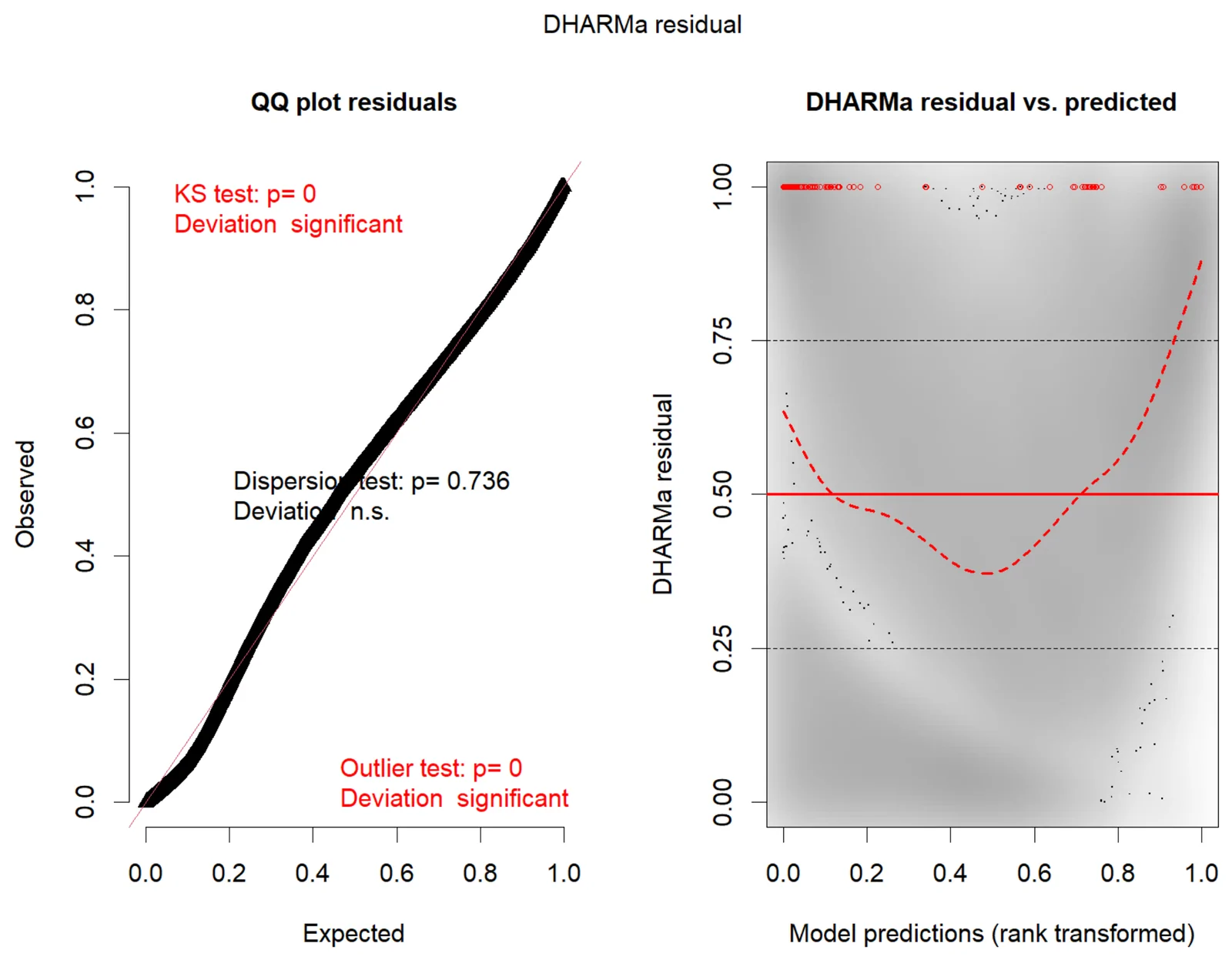
DHARMa residual diagnostics plots for the best temperature model with NB2 response. **Left** panel: QQ/uniformity plot. Thin red line represents perfect fit to the Uniform distribution. **Right** panel: scaled residuals vs. rank-transformed predicted values. The gray shading represents the density of residual observations. Horizontal red line at 0.5: expected mean residual under a correctly specified model. Dashed red line: smoothed estimate of the residual trend across fitted values. Red dots: residuals identified as simulation outliers.

Figure 16 shows the DHARMa residual plots for the best temperature model (Model 6) fitted with a negative binomial (NB2) distribution. The left panel presents a quantile–quantile (QQ) plot of the simulated residuals against the expected uniform distribution, where each point represents a scaled residual and the red diagonal line indicates the theoretical expectation under correct model specification; systematic deviations from this line indicate potential model misspecification. The right panel displays residuals versus predicted values (rank-transformed), where the residuals should be uniformly distributed and centered around 0.5 with no discernible pattern under a well-specified model. The dashed red curve is a smoothed estimate of the residual trend across fitted values. The horizontal solid red line at 0.5 represents the expected mean residual under a correctly specified model. The gray shading represents the density of residual observations, with darker regions indicating a greater concentration of points. A notable feature is the presence of several residuals equal to 1 labeled as red dots, indicating that the corresponding observed values lie at or beyond the upper tail of the simulated distribution (i.e., nearly all simulated responses are smaller than the observations), suggesting that the model may underestimate extreme values. The observed curvature of the smoothed residual trend suggests systematic deviations from the expected uniform residual distribution across the prediction range, which is consistent with the significant KS test (*p* = 0) and outlier test (*p* = 0) reported in the left panel.

**Figure 17:**
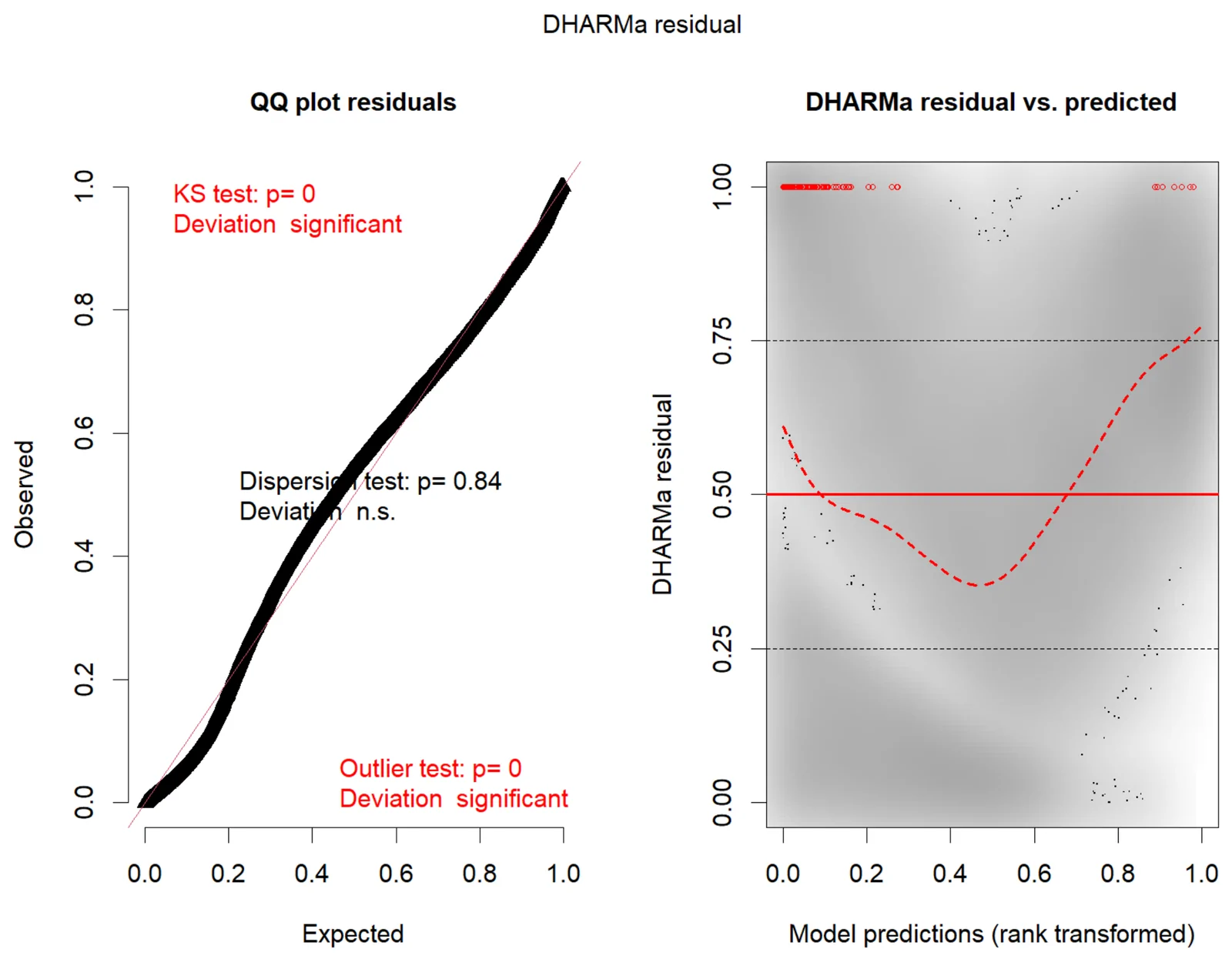
DHARMa residual diagnostics plots for the best heat index model with NB2 response. **Left** panel: QQ/uniformity plot. Thin red line represents perfect fit to the Uniform distribution. **Right** panel: scaled residuals vs. rank-transformed predicted values. The gray shading represents the density of residual observations. Horizontal red line at 0.5: expected mean residual under a correctly specified model. Dashed red line: smoothed estimate of the residual trend across fitted values. Red dots: residuals identified as simulation outliers.

Analogous to Figure 16, Figure 17 presents the DHARMa residual plots for the best heat index model (Model 5) using the NB2 distribution family.

Similar results are observed for the heat index model (Figure 17). The diagnostic tests yield consistent conclusions for both models. The Kolmogorov–Smirnov (KS) test for uniformity is significant, indicating that the residuals deviate from the expected uniform distribution. In contrast, the dispersion test is not significant, suggesting that both models adequately account for overdispersion. However, the outlier test is significant, indicating an excess number of observations falling outside the simulated range, which is consistent with a lack of fit in the upper tail of the distribution.

Taken together, these diagnostics suggest that, although the negative binomial models successfully address overdispersion, they fail to adequately capture extreme observations and possibly other structural features of the data. The significant deviation from uniformity further indicates residual misspecification, potentially arising from unmodeled nonlinear effects, temporal dependence, or missing covariates. While the NB2 specification provides an improvement over the Poisson model, additional model refinement is required to better represent the distributional characteristics of heat-related emergency department visit counts.

## 5. Discussion

This study examined the relationship between extreme heat indicators and emergency department visits (EDV) for heat-related illnesses across the ten U.S. Health and Human Services regions over the period 2018–2025. By combining seasonal analysis with generalized linear mixed-effects models, we provide insights into the temporal dynamics, regional variability, and predictive performance of climate–health relationships at a national scale.

A novel contribution of this study is the quantification of regional differences in peak timing across HHS regions and the demonstration that EDV seasonality is not perfectly synchronized with either temperature or heat index seasonality.

The average EDV peak occurs around day 194 of the year, while maximum temperature and heat index peak later, around days 200 and 203, respectively. The observed differences of approximately 6 days for temperature and 9 days for heat index, suggest that EDV begin increasing before environmental variables reach their maximum values. This pattern aligns with previous findings reported in the literature, where increased heat-related morbidity precedes peak temperature conditions, likely reflecting early exposure effects and population vulnerability conditions. Our findings are also consistent with those of [15], who demonstrated that populations become acclimatized over the course of a season, leading to stronger health effects during early-season heat events than during comparable temperatures later in summer.

Multiple studies have reported increased hospitalizations and emergency department utilization during periods of extreme heat and have documented substantial geographic heterogeneity in heat-related risks [16,17,18,19]. Our findings are consistent with this literature and further show that heat index provides slightly better predictive performance than temperature alone.

A key result of this analysis is the immediate heat effect on the response variable EDV. The cross-correlation analysis found that, in most regions, the strongest association between EDV and environmental variables occurred at lag 0, indicating an immediate response to heat exposure. This finding suggests that heat-related health impacts are largely contemporaneous with exposure, emphasizing the importance of real-time monitoring and rapid response at the spatial and temporal scales examined in this study. This result agrees with many epidemiological studies that report the strongest heat-related morbidity effects occurring on the same day or within one day of exposure. For example, ref. [20] used distributed lag nonlinear models across nearly 3000 U.S. counties and found that much of the excess risk associated with extreme heat occurred immediately following exposure, with the largest effects concentrated at short lags. Similarly, recent emergency department studies using distributed lag models have shown that the effect of temperature on Emergency Department utilization is often strongest at lag 0, with smaller contributions from subsequent days [21].

While lag-zero correlations dominated the present analysis, delayed health impacts extending over multiple days cannot be ruled out. Future studies using distributed lag or distributed lag nonlinear modeling frameworks could provide a more detailed characterization of the temporal dynamics of heat-related healthcare utilization.

Regional heterogeneity was also observed in the relationship between temperature and EDV. This finding aligns closely with national studies reporting geographic heterogeneity in heat-health relationships. Ref. [20] found stronger heat-related ED risks in the northeastern U.S. and regions with continental climates than in other parts of the country. More recently, analyses of Medicare-Medicaid beneficiaries demonstrated substantial regional variation in the association between extreme heat and all-cause emergency department visits across major U.S. climate regions [22]. Our results extend this literature by explicitly modeling regional heterogeneity through random effects and demonstrating that region-specific temperature associations improve predictive performance. In contrast, the best-performing heat index model suggested that the relationship between heat index and EDV is more consistent across regions, indicating that the heat index may capture more uniform physiological responses to heat exposure across regions reducing the need for region-specific slopes. However, its performance is not substantially different from that of the other models that use the heat index as a predictor. Previous studies have generally found strong associations between both temperature-based and heat-stress metrics and heat-related morbidity but have not reached a consensus regarding which metric is superior nationally. Many large U.S. analyses continue to use ambient air temperature as the primary exposure because of data availability and interpretability.

Model comparison revealed a trade-off between predictive accuracy and distributional fit. While Poisson models achieved lower mean squared error (MSE), indicating better predictive performance, negative binomial (NB2) models provided improved goodness of fit by accounting for overdispersion in the data. The lower out-of-sample MSE achieved by the Poisson models indicates superior predictive accuracy for the conditional mean response. However, because the data exhibited overdispersion, the NB2 models provided a better representation of the underlying count distribution and remain preferable for inference. While the Poisson model provided the best predictive accuracy in several analyses, this finding should not be interpreted as implying inferential superiority; when overdispersion is present, the Negative Binomial model remains more appropriate for estimation and uncertainty quantification because it explicitly accounts for extra-Poisson variation.

The intra-class correlation coefficient (ICC) analysis provides additional insight about the sources of EDV variability. Unadjusted ICC values were relatively low (approximately 5–10% for the Poisson family and 4% for the NB2 family), indicating that region-to-region differences alone explain a limited portion of total sources of variability.

After adjusting for seasonal and environmental covariates, most of the remaining unexplained variability is attributed to regional differences in the Poisson case. The adjusted ICC values for the Poisson models are very close to 1. This indicates that, after accounting for seasonal and meteorological covariates, nearly all of the remaining unexplained variability is attributable to between-region differences. In other words, the residual variation becomes highly structured at the regional level, with very little within-region variability remaining once covariates are included. For the NB2 model case, additional variability is accounted for by overdispersion, and only a moderate fraction of the remaining unexplained variability (11–14%) after fixed effects adjustment, is attributed to regional differences. The NB2 ICC estimates (≈0.11–0.14) are likely the more meaningful measures of clustering because they account for overdispersion. The near-unity Poisson ICC values arise from the restrictive Poisson variance assumption and should not be interpreted as indicating that nearly all EDV variability occurs between HHS regions.

Residual diagnostics indicated remaining model misspecification despite accounting for overdispersion, particularly for the most extreme EDV observations. These findings suggest that additional predictors and more flexible model structures may be required to better represent rare but high-impact events.

Unlike the distributed lag nonlinear modeling (DLNM) framework commonly used in heat-health epidemiology, the present study employed generalized linear mixed models to emphasize prediction and quantification of regional heterogeneity. DLNMs are particularly well suited for estimating nonlinear exposure-response relationships and delayed temperature effects, whereas the mixed-effects approach allows direct modeling of region-specific variation through random effects. Because our lag-detection analysis indicated that the strongest associations occurred at lag 0, a distributed lag structure was not included. Consequently, the proposed models provide a parsimonious framework for prediction while accounting for geographic variability in heat-health relationships across HHS regions, although they may be less flexible than DLNMs in capturing complex nonlinear and delayed responses to heat exposure.

Several important limitations of this study should be acknowledged. First, the analysis is limited by the availability of EDV data beginning in 2018, restricting the evaluation of long-term trends and variability. Second, the use of HHS regions as the spatial unit of analysis may obscure sub-regional heterogeneity and localized heat-health relationships. Although this spatial scale was dictated by the availability of the EDV data, it masks considerable within-region heterogeneity in climate, urbanization, population characteristics, socioeconomic conditions, healthcare access, and adaptive capacity. Consequently, the estimated associations represent average regional relationships and should not be interpreted as directly reflecting county, city, or individual-level heat-health responses. Third, although vulnerability-related variables were not retained because they did not achieve statistical significance and better goodness of fit, this result may be attributable to limitations in data resolution rather than a lack of underlying associations. Future studies incorporating stratified analyses by sex, age, socioeconomic status, and other vulnerability indicators may provide further insight into differential population responses to heat exposure.

An additional limitation of this study is the ecological inference limitation. Because the analysis was conducted at the HHS regional level, the estimated associations represent relationships between aggregated regional measures of heat exposure and health outcomes. Therefore, the findings are subject to ecological fallacy, whereby associations observed for groups may not reflect relationships at the individual, community, or county level. Considerable heterogeneity in climate, urbanization, socioeconomic conditions, healthcare access, and adaptive capacity exists within each HHS region and is not fully captured by regional averages. Accordingly, the results should be interpreted as describing broad regional trends rather than local heat-health relationships. Future work incorporating county-level health outcome data and spatially explicit modeling approaches could better characterize within-region variability and strengthen causal interpretation.

Another limitation is the assumption of a linear relationship between heat indicators and emergency department visits. Previous studies have shown that heat-health associations are often nonlinear, with threshold effects and more rapid increases in risk at extreme temperatures. Therefore, the linear models used in this study should be viewed as providing average associations over the observed exposure range rather than fully characterizing the underlying exposure-response relationship. If nonlinear relationships are present, the estimated effects may mask important variations in risk across different heat conditions. Future research using Generalized Additive Models, spline-based methods, or Distributed Lag Nonlinear Models could better capture potential thresholds, nonlinear exposure-response patterns, and delayed health effects.

## 6. Conclusions

In summary, this study demonstrates that environmental heat indicators, particularly heat index, are valuable predictors of heat-related emergency department visits across the United States. Models incorporating temperature or heat index substantially outperform purely seasonal models, underscoring the importance of including environmental drivers in predictive frameworks.

The finding that EDV peaks occur earlier than the peaks of temperature and heat index has important public health implications. It suggests that interventions such as heat-health warning systems and preparedness strategies should be implemented prior to the highest levels of heat exposure, rather than in response to peak conditions.

From a methodological standpoint, mixed-effects modeling provides a useful framework for capturing both shared seasonal trends and regional variability. However, further work is needed to address model limitations related to overdispersion, residual structure, and extreme responses. Although the proposed models demonstrated satisfactory predictive performance overall, residual misspecification remained, particularly for the largest observed emergency department visit counts. This limitation suggests that predictive accuracy may decline during the most extreme heat events, when health system demands are highest and accurate forecasts are most critical for preparedness and resource allocation. Future research should therefore focus on improving model performance under extreme conditions, including the incorporation of nonlinear and threshold-based relationships that may better capture rare but impactful events.

Future research should focus on improving spatial and temporal resolution of both health and environmental data, expanding the temporal coverage of EDV records, and enhancing access to integrated datasets. Improved data availability will be critical for better understanding lagged effects, vulnerability factors, and regional disparities in heat-related health outcomes.

Overall, this study contributes to the growing evidence linking extreme heat to increased healthcare demand and provides a basis for developing regionally adaptive public health strategies aimed at mitigating the impacts of a changing climate on population health.

## Figures and Tables

**Table 1:** Adjusted and unadjusted ICCs for best temperature and heat index models.

Covariate	Adjusted ICC		Unadjusted ICC	
Model Family	Poisson	NB2	Poisson	NB2
Temperature (2018–2022)	1.000	0.115	0.086	0.040
Temperature (2018–2025)	1.000	0.137	0.102	0.044
Heat index (2018–2022)	0.999	0.106	0.058	0.036

## Data Availability

Data used in this study is available in the public domains: Centers for Disease Control and Prevention’s (CDC’s) National Environmental Public Health Tracking Network (https://ephtracking.cdc.gov/DataExplorer/, accessed on 24 February 2026) and the Physical Sciences Laboratory of the National Oceanic and Atmospheric Administration (NOAA) (https://psl.noaa.gov/data/gridded/data.cpc.globaltemp.html, accessed on 4 March 2025).

## Abbreviations

The following abbreviations are used in this manuscript:

CDC	Centers for Disease Control and Preventions
EDV	Emergency Department Visits
HHS	Health and Human Services
HI	Heat Index
NOAA	National Oceanic and Atmospheric Administration

## Appendix A

**Table A1:** Seasonality metrics for EDV: $P_{T}$: Peak timing (number of days relative to the start of the year); $\sigma_{P_{T}}$: $P_{T}$ Standard Error; γ: Amplitude (Degrees Celsius); $\sigma_{\gamma }^{2}$: γ Variance.

Region	Peak Timing ($P_{T}$)	$\sigma_{P_{T}}$	Amplitude (γ)	$\sigma_{\gamma }^{2}$
Region 1	192.9	0.9617	4.596	0.0835
Region 2	191.8	0.8782	4.791	0.0812
Region 3	192.4	0.7399	3.964	0.0314
Region 4	196.2	0.5312	2.299	0.0025
Region 5	187.5	0.9239	3.451	0.0341
Region 6	197.5	0.5739	2.487	0.0036
Region 7	193.2	0.7998	3.131	0.0169
Region 8	194.3	0.5347	3.607	0.0116
Region 9	198.8	0.5682	2.887	0.0056
Region 10	195.4	1.338	4.479	0.1396

**Table A2:** Seasonality metrics for temperature: $P_{T}$: Peak timing (number of days relative to the start of the year); $\sigma_{P_{T}}$: $P_{T}$ Standard Error; γ: Amplitude (Degrees Celsius); $\sigma_{\gamma }^{2}$: γ Variance.

Region	Peak Timing ($P_{T}$)	$\sigma_{P_{T}}$	Amplitude (γ)	$\sigma_{\gamma }^{2}$
Region 1	202.5	0.4661	14.08	0.0127
Region 2	201.7	0.4995	13.17	0.0128
Region 3	199.1	0.5592	11.94	0.0132
Region 4	197.8	0.5717	9.314	0.0084
Region 5	200.5	0.4539	15.06	0.0138
Region 6	198.7	0.5521	10.64	0.0102
Region 7	200.8	0.5706	14.01	0.0189
Region 8	202.4	0.4612	15.29	0.0147
Region 9	203.0	0.4323	11.85	0.0078
Region 10	197.1	0.3158	15.23	0.0068

**Table A3:** Seasonality metrics for heat index: $P_{T}$: Peak timing (number of days relative to the start of the year); $\sigma_{P_{T}}$: $P_{T}$ Standard Error; γ: Amplitude (Degrees Celsius); $\sigma_{\gamma }^{2}$: γ Variance.

Region	Peak Timing ($P_{T}$)	$\sigma_{P_{T}}$	Amplitude (γ)	$\sigma_{\gamma }^{2}$
Region 1	207.4	0.5505	13.76	0.017
Region 2	204.7	0.5467	14.45	0.0185
Region 3	202.4	0.6004	13.69	0.02
Region 4	200.4	0.6351	10.98	0.0144
Region 5	201.7	0.5132	16.05	0.0201
Region 6	197.1	0.6368	12.24	0.018
Region 7	200.3	0.5618	16.39	0.0251
Region 8	203.1	0.4388	14.53	0.012
Region 9	207.9	0.5274	10.51	0.0091
Region 10	205.2	0.5231	11.58	0.0109
